# Seasonal variation of secondary metabolites in nine different bryophytes

**DOI:** 10.1002/ece3.4361

**Published:** 2018-08-22

**Authors:** Kristian Peters, Karin Gorzolka, Helge Bruelheide, Steffen Neumann

**Affiliations:** ^1^ Leibniz Institute of Plant Biochemistry, Stress and Developmental Biology Halle Germany; ^2^ Institute of Biology/Geobotany and Botanical Garden Martin Luther University Halle Wittenberg Halle Germany; ^3^ German Centre for Integrative Biodiversity Research (iDiv) Halle‐Jena‐Leipzig Leipzig Germany

**Keywords:** biochemistry, bryophytes, chemotaxonomy, ecology, ecometabolomics, environment, liverworts, mosses, phylogeny

## Abstract

Bryophytes occur in almost all land ecosystems and contribute to global biogeochemical cycles, ecosystem functioning, and influence vegetation dynamics. As growth and biochemistry of bryophytes are strongly dependent on the season, we analyzed metabolic variation across seasons with regard to ecological characteristics and phylogeny. Using bioinformatics methods, we present an integrative and reproducible approach to connect ecology with biochemistry. Nine different bryophyte species were collected in three composite samples in four seasons. Untargeted liquid chromatography coupled with mass spectrometry (LC/MS) was performed to obtain metabolite profiles. Redundancy analysis, Pearson's correlation, Shannon diversity, and hierarchical clustering were used to determine relationships among species, seasons, ecological characteristics, and hierarchical clustering. Metabolite profiles of *Marchantia polymorpha* and *Fissidens taxifolius* which are species with ruderal life strategy (R‐selected) showed low seasonal variability, while the profiles of the pleurocarpous mosses and *Grimmia pulvinata* which have characteristics of a competitive strategy (C‐selected) were more variable. *Polytrichum strictum* and *Plagiomnium undulatum* had intermediary life strategies. Our study revealed strong species‐specific differences in metabolite profiles between the seasons. Life strategies, growth forms, and indicator values for light and soil were among the most important ecological predictors. We demonstrate that untargeted Eco‐Metabolomics provide useful biochemical insight that improves our understanding of fundamental ecological strategies.

## 1 INTRODUCTION

There are approx. 20,000 bryophyte species known to science. Bryophytes are classified into three major groups: liverworts (“hepatics”, *Marchantiophyta*), mosses *s. str*. (“musci”, *Bryophyta*), and hornworts (*Anthocerophyta*) (Bowman et al., 2017; Goffinet & Shaw, 2009; Qiu et al., 2006; Shaw, Szovenyi, & Shaw, 2011). They occur in nearly every land ecosystem (Vanderpoorten & Goffinet, 2009).

Bryophytes contain many unique chemical compounds with high biological and ecological relevance (Asakawa, Ludwiczuk, & Nagashima, 2013a). Due to unique oil bodies, liverworts are biochemically very distinctive from other mosses. Secondary metabolites in oil bodies are mostly composed of lipophilic terpenoids, abundant (bis‐)bibenzyls, and small aromatic compounds (Asakawa et al., 2013a). Liverworts represent a phylogenetic group of plants that were the first colonizers of land; thus, they share many biochemical features of both algae and land plants (Bowman et al., 2017). It has been acknowledged that there must have been many biochemical innovations involved during evolution from water to land (He, Sun, & Zhu, 2013; Suire et al., 2000). Even though oil bodies in *M. polymorpha* are usually restricted to only few vegetative cells of the thallus, relative number of oil bodies has been correlated to growth conditions, availability of nutrients, level of plant‐herbivory, and biodiversity (Tanaka et al., 2016). The compounds unique to liverworts are involved in many biotic interactions and act as defense to herbivory (Asakawa, Ludwiczuk, & Nagashima, 2013b).

Despite the fact that the majority of bryophytes (approx. 14,000 species) belong to the group of mosses (Bryophyta), fewer compounds have been characterized in mosses than in liverworts. Mosses contain terpenoids; benzoic, cinnamic, and phthalic acid derivatives; coumarins; and some nitrogen‐containing aromatic compounds, which sometimes are structurally similar to those found in vascular plants (Asakawa et al., 2013a).

As secondary metabolite profiles are similar among phylogenetically closely related species (Maksimova, Klavina, Bikovens, Zicmanis, & Purmalis, 2013; Wink, 2003; Wu, 1992), metabolomics can also be used to support phylogenies based on genetic markers, for example, to find marker compounds to assist current phylogenetic classifications, to discriminate several ecotypes of bryophyte species, or even to propose new chemical taxonomic markers (Heinrichs, Anton, Gradstein, & Mues, 2000; Pejin et al., 2010; Rycroft, Heinrichs, Cole, & Anton, 2001).

Several hundred new compounds have been isolated from bryophytes in recent years. Species produce secondary metabolites as a defense against mechanical damage, environmental stress, herbivores, and pathogens, as well as to capture and conserve resources (Cornelissen, Lang, Soudzilovskaia, & During, 2007). However, there is still a knowledge gap with regard to the ecological relevance of compounds (Asakawa et al., 2013b).

Bryophytes exhibit allelopathic interactions with other organisms by releasing allelochemicals. For example, as some slugs feed on bryophytes, mosses such as *Dicranum scoparium* have evolved acetylic oxylipins that act as a defense against herbivorous slugs (Boch, Prati, & Fischer, 2016; Rempt & Pohnert, 2010). Other oxylipins or related compound classes have also been found to induce defense reactions in vascular plants. In this context, several studies found both inhibition and facilitation effects of bryophytes on seed germination and seedling growth of vascular plants (Donath & Eckstein, 2010; Michel, Burritt, & Lee, 2011; Zamfir, 2000). In addition, positive and negative effects of bryophytes on species diversity have been described. As a result, the effect of bryophytes on diversity cannot be generalized as it has been found to depend on the type of habitat and environmental conditions (Ehlers, Damgaard, & Laroche, 2016; Gornall, Woodin, Jónsdóttir, & van der Wal, 2011; Hüllbusch, Brandt, Ende, & Dengler, 2016; Jeschke & Kiehl, 2008; Müller et al., 2012).

Despite their small size, bryophytes show remarkable biochemical adjustments to environmental changes (During, 1992; Klavina, 2015). For example, bryophyte species that occur as colonizers in early successional stages collect debris, store water, and deposit and solidify soil. Thus, bryophytes can reduce erosion and often act as prerequisite for establishing vascular plants by creating microhabitats (Streitberger, Schmidt, & Fartmann, 2017; Zamfir, 2000). In late successional stages in grasslands, even low bryophyte abundances can facilitate the regeneration of vascular plants by influencing nutrient retention and water cycling (Virtanen, Eskelinen, & Harrison, 2017). However, the net outcome is often depending on environmental conditions (Doxford, Ooi, & Freckleton, 2013).

There are many studies that link the abundance and the distribution of bryophytes with the environment (Aranda et al., 2014; Smith, 1982). Altitudinal gradients were often used to study the effects of seasons and environments in combination (Mateo et al., 2016; Sun et al., 2013; Wagner, Zotz, Salazar Allen, & Bader, 2013). However, there are only few studies that analyzed the biochemical responses of bryophytes to different environments or seasons. For example, studies with the liverwort *Conocephalum conicum* revealed largely different metabolite profiles of morphologically mostly indistinguishable specimen that were collected in contrasting environments (Ghani, Ludwiczuk, Ismail, & Asakawa, 2016; Ludwiczuk, Odrzykoski, & Asakawa, 2013). A different study analyzed three leafy liverwort species and found seasonal variation in antioxidant and polyphenol oxidase enzymes, as well as in the flavonoid and phenolic content (Thakur & Kapila, 2017).

Bryophytes have adopted different types of ecological strategies (During, 1992; Frisvoll, 1997) (Table 1). Grime (1977) described three basic types of life strategies for plants (the so‐called CSR triangle). Competitive species (C‐selected) show high nutrient turnover, large relative growth rates, morphological plasticity, a long life span, and usually low reproduction (During, 1992). They are typically found in late successional habitats. The S‐selected group consists of stress‐tolerant species that are slowly growing, have a conservative nutrient uptake, and are usually found in habitats that have abiotic constraints, for example, limited resource availability. Many ruderal species are R‐selected and have traits related to fast growth, rapid nutrient uptake, high reproduction, and a short life span (Ayres, van der Wal, Sommerkorn, & Bardgett, 2006). They are usually found in early successional habitats and are quickly overgrown by competitors. There are also many species with intermediary strategies, especially epiphytic and epilithic bryophytes (During, 1992; Frisvoll, 1997).

**Table 1:** Life history characteristics of the bryophytes used in the study were collected from the literature

Code	Species	Family	Type	Growth form	Habitat type	Substrate	Life strategy	Gametangia distribution	Mean spore size [μm]	Sexual reproduction frequency	Light index	Temperature index	Continentality index	Moisture index	Reaction index	Nitrogen index	Life‐form index
Brarut	*Brachythecium rutabulum*	Brachytheciaceae	Pleurocarpous	Mat	Woods, Shrubs	Soil, Firm rocks	Perennial stayer competitive	Autoicous	20	Common	5	5	5	4	5	9	C,(E)
Calcus	*Calliergonella cuspidata*	Amblystegiaceae	Pleurocarpous	Mat	Meadows, Herbaceous	Soil, Turf	Perennial stayer competitive	Dioicous	20	Occasional	8	3	5	7	7	8	C
Fistax	*Fissidens taxifolius*	Fissidentaceae	Acrocarpous	Turf	Woods, Shrubs	Soil	Colonist	Autoicous	15	Occasional	5	4	5	6	7	5	H
Gripul	*Grimmia pulvinata*	Grimmiaceae	Acrocarpous	Cushion	Exposed Rocks	Firm rocks	Pioneer	Autoicous	10	Very common	8	5	5	1	7	7	C
Hypcup	*Hypnum cupressiforme* s. l.	Hypnaceae	Pleurocarpous	Mat	Woods, Shrubs	Dead wood, Bark	Perennial stayer stress‐tolerant	Dioicous	14	Common	5	5	5	4	4	8	C, E
Marpol	*Marchantia polymorpha* s. l.	Marchantiaceae	Liverwort	Thalloid	Ruderal, Banks	Soil, Loose rocks	Colonist	Dioicous	14	Common	8	5	5	6	5	8	T
Plaund	*Plagiomnium undulatum*	Mniaceae	Acrocarpous	Dendroid	Woods, Shrubs	Soil	Long‐lived shuttle	Synoicous	28	Rare	4	3	5	6	6	7	H, C
Polstr	*Polytrichum strictum*	Polytrichaceae	Acrocarpous	Turf	Woods, Shrubs	Turf, Soil	Perennial stayer competitive	Dioicous	16	Common	8	2	6	6	1	4	H
Rhysqu	*Rhytidiadelphus squarrosus*	Hypnaceae	Pleurocarpous	Mat	Meadows, Herbaceous	Soil	Perennial stayer competitive	Dioicous	19	Rare	7	3	6	6	5	7	C

Note. Family and type are based on the taxonomic classification found in Smith (1990, 2004); The characteristics “growth form,” “habitat type,” and “substrate” were added from the tables in Urmi (2010); “life strategy” is based on the classification of During (1992) and was added from tables in Frisvoll (1997); “spore size,” “gametangia distribution,” and “sexual reproduction frequency” were collected from Smith (1990, 2004); Ellenberg indicator values (light, temperature, continentality, moisture, reaction, nitrogen, and life‐form indices) were added from Urmi (2010).

Many morphological and physiological relationships have been described to be correlated with these plant strategy types (e.g., leaf area, growth, and photosynthesis), including the capabilities of bryophytes that drive biogeochemical processes (Caccianiga, Luzzaro, Pierce, Ceriani, & Cerabolini, 2006; Cornelissen et al., 2007; Grime, Rincon, & Wickerson, 1990). Linking metabolites to plant strategy theory contributes to a mechanistic understanding of how bryophytes are able to, for example, tolerate desiccation biochemically and are still able to grow under dry and cool conditions (Grime et al., 1990).

Recent advances in analytical methods (e.g., liquid chromatography coupled with mass spectrometry—LC/MS) allow to simultaneously measure most semipolar metabolites of an organism at once in an untargeted way (without specifically targeting some known compounds). In an ecological context, this is known as Eco‐Metabolomics (Hall, 2006; Sardans, Peñuelas, & Rivas‐Ubach, 2011). When compared to typical biochemical experiments, where plants are usually grown under controlled conditions in glasshouses or growth chambers, in Eco‐Metabolomics, metabolite profiles are typically acquired from wild plant species in their natural environment (van Dam & van der Meijden, 2011; Rivas‐Ubach et al., 2016; Sardans et al., 2011). As a result, experiment designs are more complex and metabolite profiles are expected to be highly variable.

Discovering patterns in the metabolite profiles can reveal new ecological and biogeochemical relationships as the biochemistry of bryophytes is related to the environment, climate, and biotic interactions (Sardans et al., 2011). For example, metabolite profiling of higher plants grown in field plots showed that resource limitation results in decreased performance of small‐statured herbs with increasing species diversity (Scherling, Roscher, Giavalisco, Schulze, & Weckwerth, 2010). Multivariate statistical methods such as principal components analysis (PCA) allow to discriminate species based on their metabolite profiles. Furthermore, profiles can also be used to discriminate species that were grown in different environments or had a history of different ecological interactions (van Dam & van der Meijden, 2011; Hall, 2006; Jones et al., 2013).

Studying the biochemistry of bryophytes is often targeting the discovery of novel potentially active compounds and natural product chemistry (Asakawa et al., 2013a). We have found only a few studies in the literature that performed untargeted metabolomics analyses (LC/MS, GC/MS, NMR) with bryophytes, and none that were performed in an ecological context (e.g., Erxleben, Gessler, Vervliet‐Scheebaum, & Reski, 2012; Klavina, 2015; Pejin et al., 2010; Rycroft et al., 2001).

In this study, we introduce an integrative Eco‐Metabolomics approach to connect biochemistry with ecology using bioinformatics methods (Hall, 2006; Sardans et al., 2011). The aims of this study are as follows: (a) to investigate metabolic differences between species as explained by ecological characteristics, in particular, with regard to the CSR life strategy types; (b) to determine biochemical differences in species profiles with regard to the seasons; (c) to find out how the metabolomes of the bryophytes reflect their phylogeny; and (d) to present a reproducible bioinformatic workflow that can be reused by other subsequent Eco‐Metabolomics studies.

## 2 MATERIALS AND METHODS

### 2.1 Field campaign and sampling

Samples of the nine moss species, *Brachythecium rutabulum* (Hedw.) Schimp., *Calliergonella cuspidata* (Hedw.) Loeske, *Fissidens taxifolius* Hedw., *Grimmia pulvinata* (Hedw.) Sm., *Hypnum cupressiforme* Hedw. *s.l*., *Marchantia polymorpha* L., *Plagiomnium undulatum* (Hedw.) T.J. Kop., *Polytrichum strictum* Menzies ex Brid., and *Rhytidiadelphus squarrosus* (Hedw.) Warnst., were collected in the Botanical Garden of Martin Luther University Halle‐Wittenberg, Germany (see Supporting Information Figure S4 for photographs of the species). Sampling was performed in summer (2016/08/08), autumn (2016/11/09), winter (2017/01/27), and spring (2017/05/11) under stable weather conditions with sunshine at least 2 days prior to sampling and during sampling. Sampling was conducted between 13:00 and 15:00.

Three composite samples of different individuals of each species were taken in each season, leading to a total of 3 × 9 × 4 = 108 samples. Only aboveground parts of the moss gametophytes were taken for sampling. Visible archegonia or antheridia, sporophytes, and any belowground parts were removed with a sterile tweezer before sampling. The gametophytic moss parts were put in Eppendorf tubes and were frozen instantly on dry ice. Life strategies and other life characteristics were collected from the literature (Table 1).

### 2.2 Biochemical protocol

Frozen moss samples were extracted according to Böttcher et al. (2009) with the following modifications: After adding 200 mg of ceramic beads (0.5 mm diameter, Roth), samples were homogenized with a tissue homogenizer (2 × 20 at 6,500 rpm; Precellys^®^ 24, Bertin Technologies, Montigny‐le‐Bretonneux, France). 1 ml ice‐cold 80/20 (v/v) methanol/water was added. Metabolites were extracted by shaking/ultrasonification/shaking for 15 min at 1000 rpm. After 15 min centrifugation at 15,000 g (rcf), 500 μl of supernatant was dried in a vacuum centrifuge at 40°C and reconstituted in 80/20 (v/v) methanol/water with the volume adjusted to the initial fresh weight of the sample to a final concentration of 10 mg fresh weight per 100 μl extract.

Ultra‐performance liquid chromatography (Waters Acquity UPLC equipped with a HSS T3 column (100 × 1.0 mm)) coupled to electrospray ionization quadrupole time‐of‐flight mass spectrometry (UPLC/ESI‐QToF‐MS) was performed using a high‐resolution MicrOTOF‐Q II hybrid quadrupole time‐of‐flight mass spectrometer (Bruker Daltonics), as described in Böttcher et al. (2009). Data were acquired in centroid mode with the following MS instrument settings for positive mode: nebulizer gas: nitrogen, 1.4 bar; dry gas: nitrogen, 6 L/min, 190°C; capillary:, 5,000 V; end plate offset: −500 V; funnel 1 radio frequency (RF): 200 Volts peak‐to‐peak (Vpp); funnel 2 RF: 200 Vpp; in‐source collision‐induced dissociation (CID) energy: 10 eV; hexapole RF: 100 Vpp; quadrupole ion energy: 3 eV; collision gas: nitrogen; collision energy: 7 eV; collision cell RF: 250 Vpp; transfer time: 70 μs; prepulse storage: 5 μs; pulser frequency: 10 kHz; and spectra rate: 3 Hz.

### 2.3 Data analyses

Raw LC/MS data were converted to the open data format mzML with the software Bruker CompassXPort 3.0.9. Raw data and metadata were published in the metabolomics repository MetaboLights as MTBLS520 (Haug et al., 2013; Peters, Gorzolka, Bruelheide, & Neumann, 2018). A computational workflow was constructed in the Galaxy workflow management system for the entire data processing pipeline of this study (Supporting information Figure S3). Required software tools, their dependencies, as well as software libraries and R packages were containerized using Docker technology to facilitate reusability on different computational environments. Source code was made publicly available on GitHub (Peters et al., 2018).

Profiles of positive mode were used for the data analyses as many important and known secondary metabolites classes in bryophytes such as flavonoids, phenylpropanoids, anthocyanins, glycosides, and previously characterized compounds such as marchantins, communins, and ohioensins ionize well in positive mode with our instrumental setup.

Detection of chromatographic peaks was performed in R with the package XCMS 1.52.0 (Tautenhahn, Bottcher, & Neumann, 2008) with two grouping factors in “phenoData”: seasons (summer, autumn, winter, spring) and species (Brarut, Calcus, Fistax, Gripul, Hypcup, Marpol, Plaund, Polstr, Rhysqu). Quality control was performed with a laboratory internal standard mix (Peters et al., 2018). As the quality control revealed no significant differences between batches, no additional corrections on the peak detection with XCMS were performed. Intensities in the peak table were log transformed before grouping. For further analysis, only features between the retention times 20 and 1,020 were kept.

Adduct annotation was performed with the package CAMERA 1.33.3 (Kuhl, Tautenhahn, Böttcher, Larson, & Neumann, 2012). A specific function getReducedPeaklist was written (method = median) that aggregates the adducts of putative compounds into a feature matrix with singular components in order to improve subsequent statistical analyses (Peters et al., 2018).

Statistical analyses were performed in R 3.4.2 using the additional packages: multtest, RColorBrewer, vegan, multcomp, multtest, nlme, ape, pvclust, dendextend, phangorn, Hmisc, gplots, and VennDiagram. A presence–absence matrix was generated from the feature matrix to determine the differences in metabolite features between the experimental factors species and season. In concordance with the “minfrac” parameter in the alignment step in XCMS, a feature was considered present if it was detected in two out of three replicates. The presence–absence matrix was used for measuring the biochemical diversity by calculating the Shannon index for each sample using the function “diversity” in vegan (Li, Heiling, Baldwin, & Gaquerel, 2016). The total number of features and the number of unique features were calculated from the presence–absence matrix accordingly.

To test factor levels for significant differences, the Tukey HSD on a one‐way ANOVA was performed post hoc using the multcomp package. Intraspecific variability of species profiles in response to the seasons was calculated with the Pearson correlation coefficient (Pearson's r) on the presence–absence matrix using the function “rcorr” in the package Hmisc. Venn diagrams were created for each species separately using the package VennDiagram.

Variation partitioning was performed using the function “varpart” in the package vegan to analyze the influence of the factors species and seasons on the metabolite profiles. Distance‐based redundancy analysis (dbRDA) using the function “capscale” with Bray–Curtis distance and multidimensional scaling in the package vegan was chosen to analyze the relation of the ecological characteristics with the species metabolite profiles (Legendre & Anderson, 1999). Ordinal and categorical ecological characteristics were transformed to the presence–absence matrices for the ordination. The model for the dbRDA was chosen with forward and backward selection using the function “ordistep” in the package vegan. Ecological characteristics were added to the plots as post hoc variables using the function “envfit” in the package vegan.

Relationships between metabolite profiles and phylogeny were analyzed by calculating Bray–Curtis distances for phylogeny and the feature matrix (function “vegdist” in vegan) followed by hierarchical clustering (function “hclust) with the complete linkage method. The chemotaxonomic plot was reordered using the function “order.optimal” (package cba), and branches of *P. strictum* and *P. undulatum* were swapped using the function “reorder” in vegan. The similarity of the two trees was determined with the normalized Robinson–Foulds metric (function “RF.dist” in package phangorn). The similarity of the distance matrices was determined with the Mantel statistic (function “mantel” in vegan).

More detailed methods and further information on the computational workflow are described in Peters et al. (2018).

## 3 RESULTS

Preprocessing of the LC/MS raw data with XCMS and CAMERA (see Materials and Methods) resulted in a feature matrix with 108 samples and 4,032 features. The corresponding data table is available in MetaboLights and was also used for biostatistics and for the components of the entire computational workflow (Peters et al., 2018).

### 3.1 Diversity of metabolite features between the species

**Figure 1:**
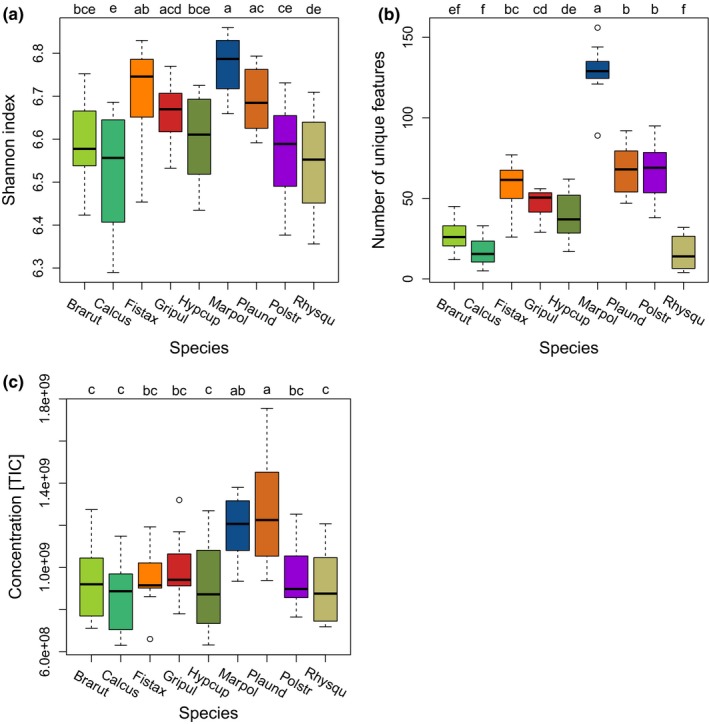
The diversity of biochemical features of the metabolite profiles of the nine bryophyte species. (a) Shannon diversity indices (H’) for the total number of features present in the species profiles. (b) Number of unique features that were exclusively present in one of the nine species. (c) Total intensities of features (= sum of total ion current) for the species. Groups for each species were calculated with performing post hoc Tukey HSD on a one‐way ANOVA. *n* = 12 for each species [Colour figure can be viewed at http://wileyonlinelibrary.com]

*Marchantia polymorpha* had significantly more biochemical features than the other species with our analytical setup (Supporting Information Table S1). In general, we observed fewer features in pleurocarpous than in acrocarpous species (Figure 1a and b, Supporting information Table S1). The relationships were also reflected in the Shannon index for the species (Figure 1a). Further, *M. polymorpha* was the species in which significantly more unique features were detected (131 ± 18) (Figure 1b). The pleurocarpous species had fewer unique features (25 ± 14) than the acrocarpous species (59 ± 17) (indicated green vs. red colors in Figure 1b; Supporting information Table S1). *M. polymorpha* and *P. undulatum* had significantly higher metabolic content per extracted gram fresh weight than the other species (Figure 1c).

### 3.2 Metabolic differences between species related to ecological characteristics

**Figure 2:**
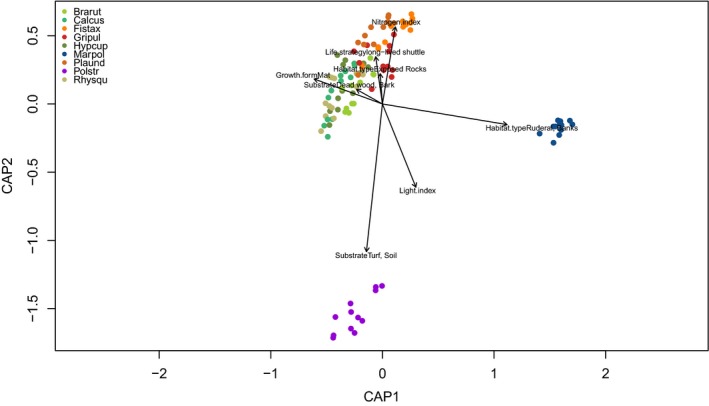
dbRDA plot of species samples (colored scores) and ecological characteristics (arrows). The length of the arrows represents the explanation power of the characteristics for the features in the matrix of metabolite profiles. The relative position of the samples to the direction of the axis describes the relationship of the sample with the characteristic. The two axes of the plot explain a total variation of 48.7% in the feature matrix. *n* = 108 samples [Colour figure can be viewed at http://wileyonlinelibrary.com]

Variation partitioning revealed that species identity accounted for 33% of the variation in the feature matrix and seasonal effects for 9% (Supporting Information Figure S1). Distance‐based redundancy analysis (dbRDA) was performed to assess the relation between ecological characteristics (Table 1) and the metabolite features of the species (Figure 2). Model selection resulted in a model of eight characteristics which explained 48.7% of the variation in the species metabolite profiles (Figure 2).

Habitat type “ruderal, banks” was responsible for the separation of *M. polymorpha* in the plot. The substrate “turf” (turfs and soils characterized by low pH) was the most powerful predictor for *P. strictum* (Figure 2). The dbRDA suggested nonlinear relationships of several indicator values with the metabolite profiles of the species. Model selection included light and nitrogen index in the model (Table 1). Profiles of *F. taxifolius* and *G. pulvinata* were correlated to the “nitrogen” indicator value. Habitat type “exposed rocks” was a powerful predictor for the epilithic *G. pulvinata*, whereas profiles of *P. undulatum* were correlated to the life strategy “long‐lived shuttle”. Growth form “mat” was the main predictor for the pleurocarpous mosses (green colored scores in Figure 2).

### 3.3 Biochemical differences in species profiles with regard to the seasons

**Figure 3:**
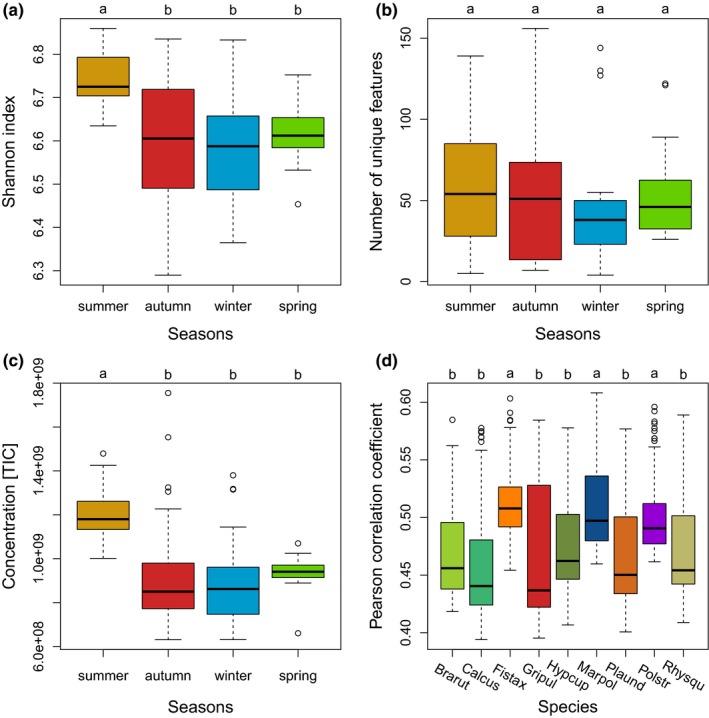
The diversity of biochemical features in the four seasons. (a) Shannon diversity indices (H’) for the total number of features present in the seasons. (b) Number of unique features that were exclusively present in one of the four seasons. (c) Total intensities of features (= sum of total ion current, TIC) per season. (d) Pearson's correlation coefficients (PCC) that show the intraspecific variability of the profiles of the species in response to the seasons. The lower the PCC values are, the more dissimilar they are, meaning higher difference in the number of features between the seasons. Groups were calculated with performing the Tukey HSD post hoc on a one‐way ANOVA. *n* = 12 for each species [Colour figure can be viewed at http://wileyonlinelibrary.com]

The total number of features present in summer (856 ± 48) was significantly higher in all species than in the seasons autumn (748 ± 108), winter (738 ± 98), and spring (762 ± 42). This was reflected by the Shannon index (Figure 3a), but not by the number of unique features in the seasons (Figure 3b). The Venn diagrams break down the proportions for each species separately (Supporting Information Figure S2). Total metabolic extracts (TIC) were also significantly higher in summer than in the other seasons (Figure 3c).

**Figure 4:**
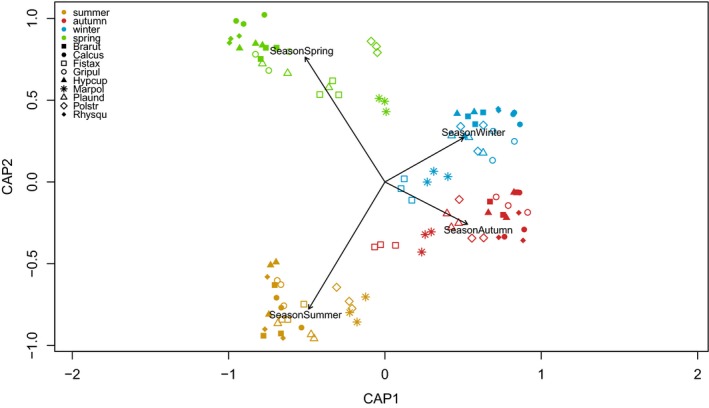
Constrained dbRDA plot of samples (colored scores) to the seasons (arrows). The length of the arrows represents the explanatory power of the season for the metabolite features. The position of the samples relative to the direction of the arrow represents the relationship of the sample with the season. The first two axes of the plot explain a total variation of 14.8% in the feature matrix. *n* = 108 samples [Colour figure can be viewed at http://wileyonlinelibrary.com]

The dbRDA using seasons as constrained variables explained 14.8% of the variation present in the feature matrix. Seasons were clearly distinct from each other (Figure 4). The dbRDA shows that metabolite profiles from autumn and winter were more similar than those from spring and summer (Figure 4). The pleurocarpous species (filled symbols in Figure 4) were less separated than the acrocarpous species. These results are in line with the number of unique features in the different species per season (Venn diagrams in Supporting Information Figure S2).

The metabolite profiles of *M. polymorpha*,* F. taxifolius,* and *P. strictum* had significantly larger Pearson Correlation Coefficients. This means that the profiles with regard to the number of features were less variable among seasons than those of the other species (Figure 3d). This lower variation among seasons is also seen in the Venn diagrams, which show the number of features that are distinct and shared between all possible combinations of the seasons and for each species separately (Supporting Information Figure S2). In contrast to the acrocarpous species, the pleurocarpous species had more distinct features between the seasons, but less shared features across the seasons.

### 3.4 Relationships of metabolite profiles and phylogeny

**Figure 5:**
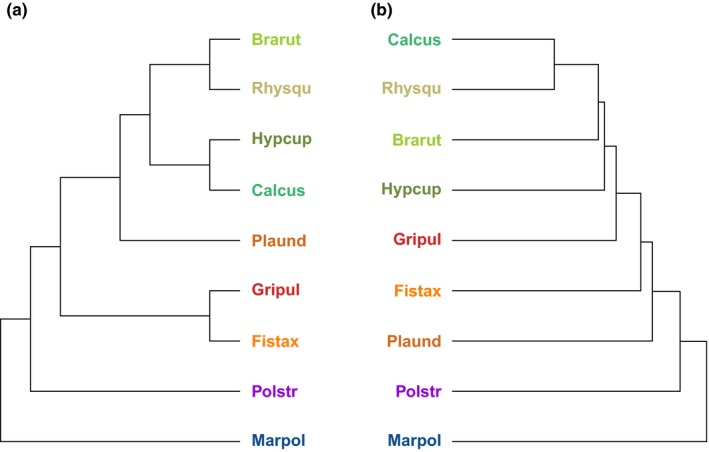
Hierarchical clustering of the bryophyte species. (a) Phylogenetic tree constructed from the phylogenetic distances of the species showing the taxonomic relationships of the bryophytes. (b) Chemotaxonomic tree resulting from hierarchical clustering of the species metabolite profiles. Height specifies the distances between the nodes [Colour figure can be viewed at http://wileyonlinelibrary.com]

In accordance with the phylogenetic tree (Figure 5a), *M. polymorpha* and *P. strictum* were identified by clustering based on metabolite features as the two most basal species with largest distances (Figure 5b). In contrast to the phylogeny, where *P. undulatum* was closer related to the group of pleurocarps than to *G. pulvinata* and *F. taxifolius*,* P. undulatum* was more dissimilar with regard to metabolite features than the other species in this clade (Figure 5b). This resulted in a higher intergroup dissimilarity of the clade.

The pleurocarpous species also formed a clade in the chemotaxonomic tree, but with different distances as in the phylogenetic tree. Comparing the two trees showed a normalized Robinson–Foulds similarity of 0.57 (where a value of 0 means total similarity and 1 means no similarity) and comparing the distance matrices of the two trees resulted in a Mantel statistics of 0.39 (Figure 5a and b).

## 4 DISCUSSION

A bioinformatic workflow was created that can be run to reproduce the results from this study (Supporting Information Figure S3). It can be reused by Eco‐Metabolomics studies with a comparable approach and with different data. Overall, our analyses revealed strong species‐specific differences in the metabolite profiles between the seasons, which could be related to the ecology of the bryophytes.

### 4.1 Bioinformatic workflow

The Galaxy workflow management provides an easy to use graphical user interface which runs in different software environments and can be operated via a web browser (Afgan et al., 2016). Our computational workflow implements the entire data processing pipeline ranging from preprocessing the metabolite profile data to multivariate statistics (Figure S3) (Peters et al., 2018). Each analysis is represented by a dedicated module in Galaxy and can be run independently to give identical results in different software environments. More importantly, modules can be adapted to other use‐cases and reused with other metabolomics data by utilizing the code which has been made available as open source (Peters et al., 2018).

Most Eco‐Metabolomics studies relate metabolite profiles to growth, stress, environment, diversity, interactions, and even geographical regions (e.g., van Dam & van der Meijden, 2011; Fester, 2015; Sardans et al., 2011; Scherling et al., 2010; Szakiel, Pączkowski, & Henry, 2011). However, comparative studies that link ecological characteristics with metabolomics are still widely missing. A comparable methodological approach was made by Frisvad, Andersen, and Thrane (2008) who related diversity in secondary metabolite profiles of filamentous fungi to life strategies. Ivanišević, Thomas, Lejeusne, Chevaldonné, and Pérez (2011) analyzed metabolic fingerprints of sponges and linked them to metabolite diversity.

With our computational workflow, we address typical challenges in Eco‐Metabolomics by analyzing data tables (one for the metabolite feature matrix and one data matrix for the ecological characteristics) conjointly with suitable statistical methods commonly used in ecology (Legendre & Legendre, 2012). As our approach follows the FAIR guiding principles for data management and stewardship (Wilkinson et al., 2016), we facilitate the reuse of our workflow by other subsequent Eco‐Metabolomics studies.

### 4.2 Relationships of metabolite diversity and phylogeny

The liverwort *Marchantia polymorpha* had significantly higher diversity of metabolite features than the other mosses with our analytical setup. This can be explained by oil bodies which are unique to liverworts and are known to contain many specialized secondary metabolites such as flavonoids, phenylpropanoids, anthocyanins, and glycosides that deter pathogens and herbivores (Bowman et al., 2017; Suire et al., 2000; Tanaka et al., 2016). In the metabolite profiles of *M. polymorpha,* we annotated many known compounds which are described as unique to liverworts in the literature (Asakawa et al., 2013a; Peters et al., 2018). The distant metabolite profiles explain also the most basal position and the largest distance of *M. polymorpha* in chemotaxonomic clustering.

The chemotaxonomic distance of *P. strictum* may be related to recent evolutionary developments such as secondary cell structures (Ligrone, Carafa, Duckett, Renzaglia, & Ruel, 2008). For example, although lignin is already present in *M. polymorpha*, its function as desiccation protective substance is less effective than in mosses where it is embedded in secondary cell structures (Ligrone et al., 2008).

In general, the dissimilarities between the phylogenetic and the chemotaxonomic tree were likely the result of different life strategies and biochemical responses of the bryophytes to the specific conditions prevalent in the habitat and may ultimately result from the differential expression of corresponding genes (Wink, 2003). This was especially evident for *P. undulatum* and could further be explained by the large separation in the dbRDA. The branch with pleurocarpous mosses represents a relatively young phylogenetic clade which can, in part, explain the weak biochemical separation of the pleurocarpous species from the others (Shaw, Cox, Goffinet, Buck, & Boles, 2003).

### 4.3 Metabolic differences between species as explained by ecological characteristics

We identified two groups of bryophytes whose metabolite profiles were either R‐ or C‐selected (During, 1992; Grime, 1977).

The R‐selected group was composed of *M. polymorpha* and *F. taxifolius*. These species had significantly more features and were significantly less variable across seasons than the other bryophyte species. These results suggest that these species rely on only a few metabolic adjustments with regard to the seasons. The two species also have ruderal characteristics such as being adaptive to the conditions in disturbed areas, fast growth and loosely growth forms, high reproduction, and being quickly overgrown by other plants with progressing succession (Frisvoll, 1997; Grime, 1977; Hedwall, Skoglund, & Linder, 2015).

Furthermore, in ruderal habitats, there could be fewer mycorrhizal associations of bryophytes and fungi as in late successional habitats (Chapin, Walker, Fastie, & Sharman, 1994). Accordingly, for the genome of *M. polymorpha* it was found that some gene families were missing that were described to be required for successful mycorrhizal associations (Bowman et al., 2017). These findings could partly explain the relatively large inventory of different metabolites that is expressed consistently throughout the whole year.

The C‐selected group included all tested pleurocarpous species *B. rutabulum*,* C. cuspidata*,* H. cupresiforme*,* R. squarrosus,* and the epilithic species *G. pulvinata*. They had low metabolite diversity, but—more significantly—showed a high seasonal variability of metabolites and, thus, produced many different features only seasonally. Except the epilithic *G. pulvinata*, species in this group were categorized as competitive (C‐selected) in the literature (Frisvoll, 1997).

Our results suggest that species in this group are specialized to the conditions in late successional stages with regard to their biochemistry, as well as to grow in mats or cushions and to have high relative growth rates in order to withstand the competition from vascular plants (During, 1992; Hedwall et al., 2015; Virtanen et al., 2017). Producing metabolites only on demand seems to be favorable for bryophyte species in late successional stages.

*Grimmia pulvinata* was categorized as pioneer by Frisvoll (1997), and as such, it should be R‐selected. However, our metabolomic data suggest that it realizes a C‐selected strategy. When only considering rocks or stones as immediate habitat, the species is very competitive to other species as it usually grows solitary.

The metabolite profiles of *Polytrichum strictum* showed an intermediary R‐ and S‐selected strategy, whereas the profiles of *Plagiomnium undulatum* showed evidence for C‐ and S‐selection. Profiles of *P. strictum* had relatively low total number of metabolite features but a high number of unique features and made little metabolic adaptations across the seasons. By contrast, profiles of *P. undulatum* had many unique and relatively high numbers of metabolites that did change considerably between the seasons. This is in accordance with the plant strategy theory which explicitly describes transitions between the different life strategies (During, 1992; Grime, 1977). According to results of Wang, Bader, Liu, Zhu, and Bao (2017), the intermediary life strategies of *Polytrichum* and *Plagiomnium* may be explained by specialized traits related to photosynthesis and growth forms.

### 4.4 Biochemical differences in species profiles with regard to the seasons

The total number of features present in summer was significantly higher than in the other seasons in any species. This can generally be explained by biological activities that are more intense during summer (Doxford et al., 2013; Lambers, Chapin, & Pons, 2008; Rousk, Pedersen, Dyrnum, & Michelsen, 2017; Thakur & Kapila, 2017). With our experimental setup, we could not measure interactions with other organisms. However, in the literature, it is also described that ecological interactions are also more manifold in the summer season in temperate regions (Grime, 1977; Lambers et al., 2008).

Bryophytes often respond sensitively to sudden climatic changes. Hence, they are considered good indicators for environmental changes (Gignac, 2001; Gilbert, 1968). It is likely that the profiles of the bryophytes we measured during summer contained also many protective substances such as sugars or polyphenols to tolerate desiccation (Erxleben et al., 2012; Garcia, Rosenstiel, Graves, Shortlidge, & Eppley, 2016; He et al., 2013; Proctor et al., 2007). However, we suggest to use additional LC/MS‐MS or NMR to identify significant metabolite features in order to make conclusions at the mechanistic level (Sardans et al., 2011).

Our results suggest that bryophytes respond species‐specifically to different seasonal conditions. The responses of bryophytes to seasons are not only depending on their ecology and the type of life strategy (see above). They are also seemed to be determined by their phylogenetic history, as metabolite profiles of pleurocarpous species were less well distinguished from those of phylogenetically more distant acrocarpous species.

## 5 CONCLUSION

We found that seasonal changes have great impact on the biochemistry of bryophytes and that the tested bryophytes realize common as well as species‐specific biochemical adjustments to the different conditions prevalent in the seasons. We further found that metabolite profiles were driven by the particular ecological characteristics and life strategies such as growth form, light availability, nutrient supply, and pH soil value. With regard to seasonal changes, the biochemistry of bryophytes is still largely unexplored. Our results warrant further biochemical investigation of bryophytes and to study relationships with ecological characteristics, life strategies, and phylogeny. With this study, we present first evidence that bryophytes realize life strategies that follow plant strategy theory by Grime (1977) at the biochemical scale. Our results demonstrate that untargeted Eco‐Metabolomics are useful to answer fundamental questions in ecology and that the ecological strategy concepts also apply to biochemical scales.

## CONFLICT OF INTEREST

None.

## AUTHOR CONTRIBUTIONS

Kristian Peters designed the experiment, participated in field sampling and collection, performed data analysis, and wrote the first draft of the manuscript. Karin Gorzolka contributed to extraction protocol and LC/MS data acquisition. Helge Bruelheide provided advice on multivariate statistics. Steffen Neumann provided advice on the bioinformatics pipeline. All authors contributed to the final version of the manuscript.

## DATA ACCESSIBILITY

Raw Metabolite profiles, metabolite feature matrices, and metadata: MetaboLights MTBLS520 (https://www.ebi.ac.uk/metabolights/MTBLS520). Computational workflow code version 1.1: Zenodo https://doi.org/10.5281/zenodo.1284246
