# Supplementary figures and images for: Seasonal variation of secondary metabolites in nine different bryophytes
Source: Ecol Evol. 2018 Aug 22;8(17):9105–17. doi: 10.1002/ece3.4361 (PMC6157681; doi:10.1002/ece3.4361)

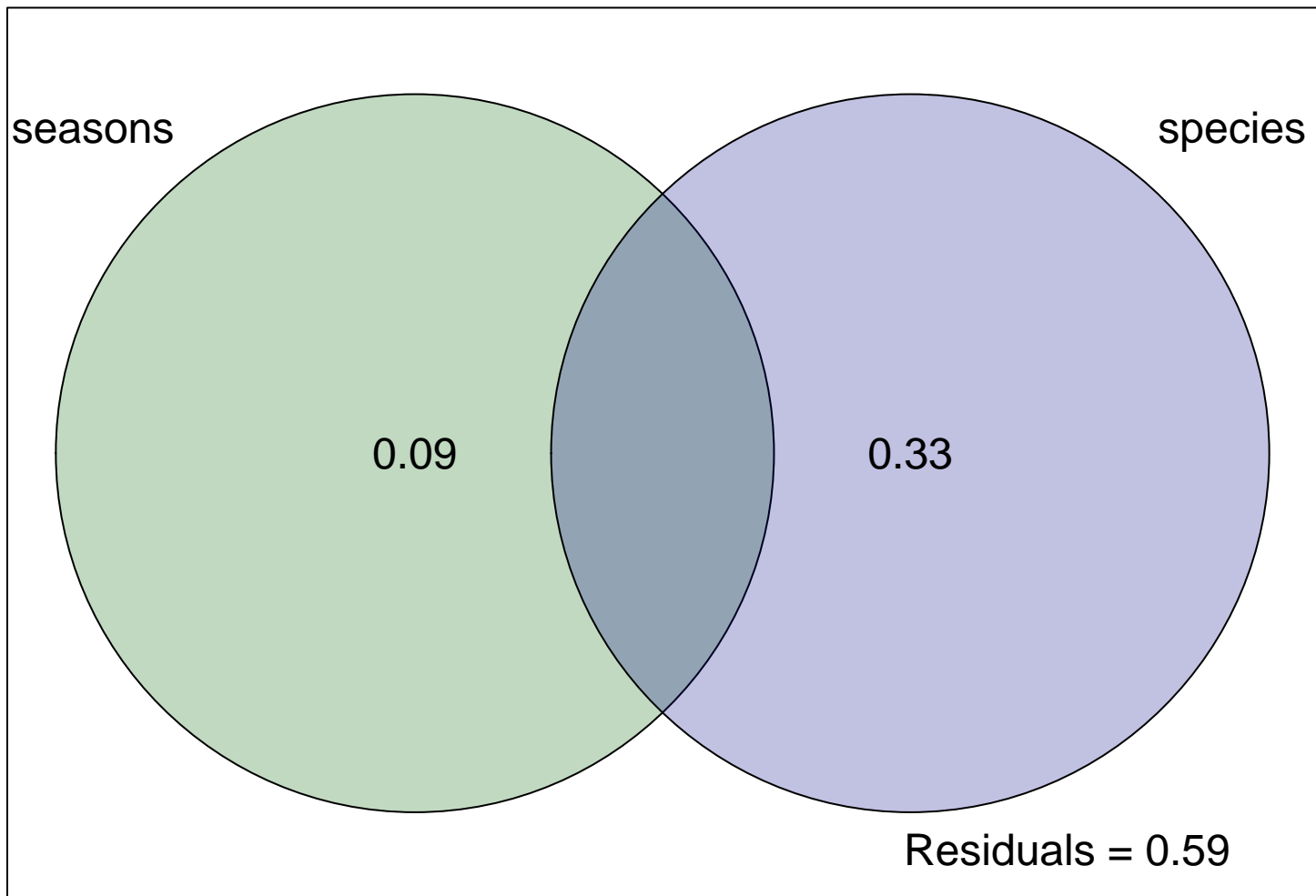

Values <0 not shown

Supplement: Supplementary file 1 [file ECE3-8-9105-s001.pdf]

**(a)**

*Marchantia polymorpha*

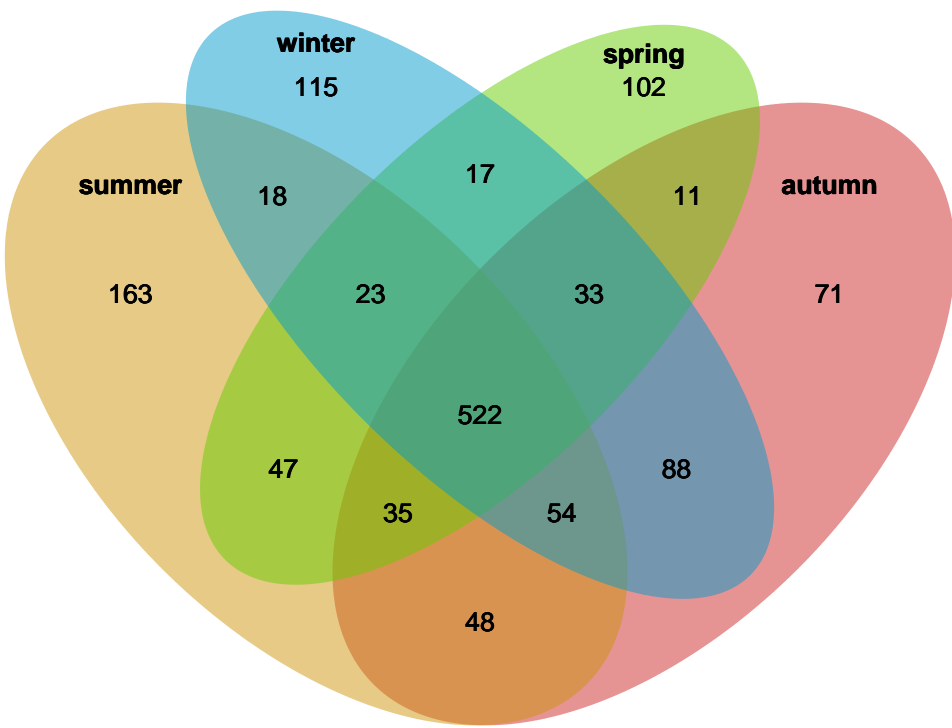

Supplement: Supplementary file 2 [file ECE3-8-9105-s002.pdf]

**(b)**

*Pleurocarpus species*

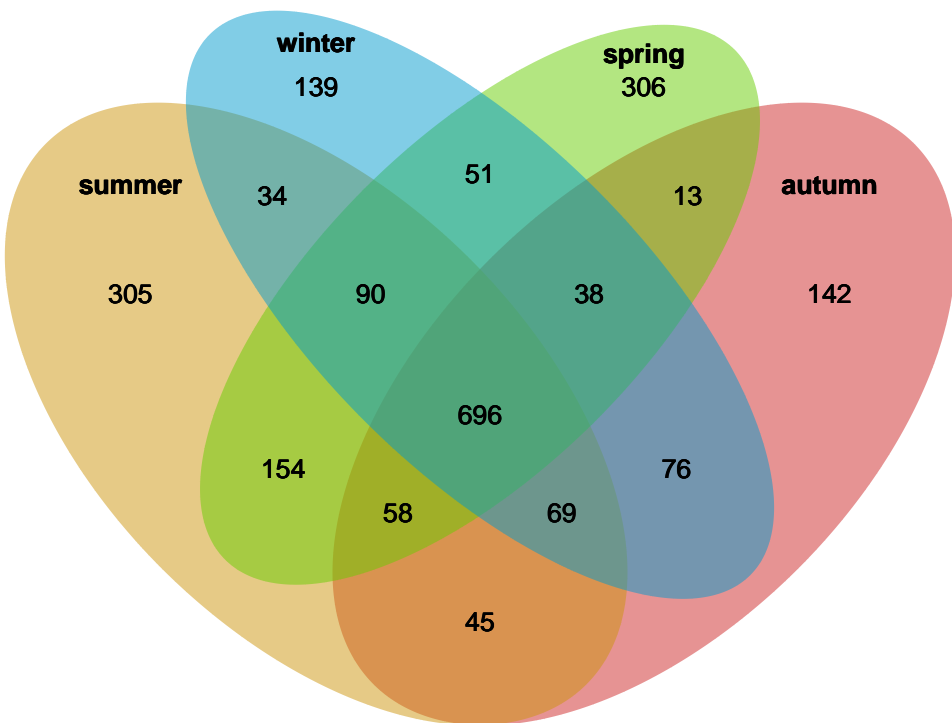

Supplement: Supplementary file 3 [file ECE3-8-9105-s003.pdf]

**(c)** *Acrocarpous species*

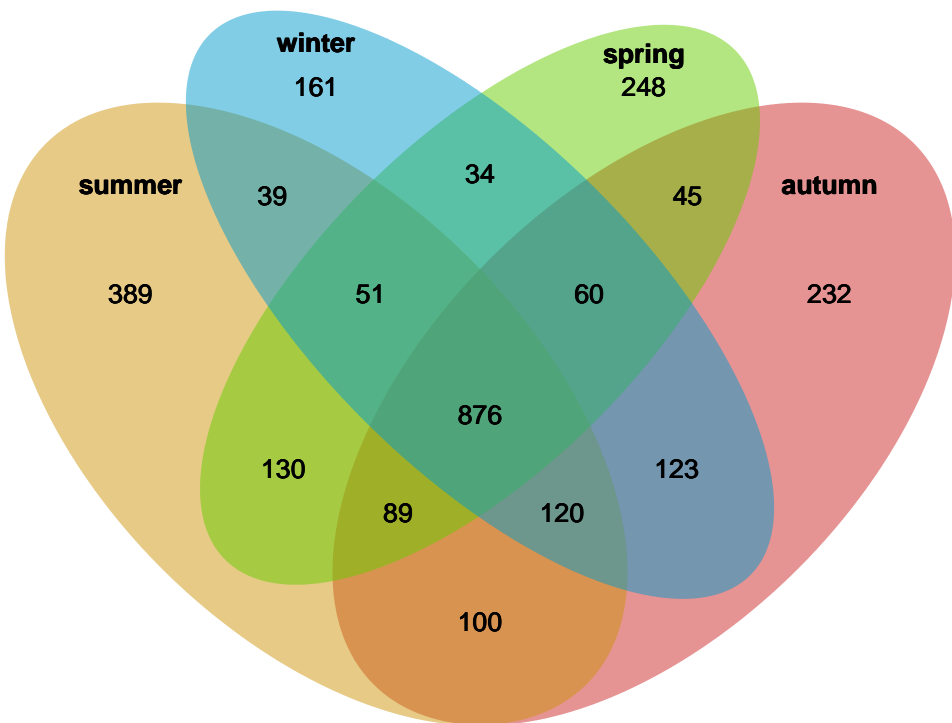

Supplement: Supplementary file 4 [file ECE3-8-9105-s004.pdf]

(d)

*Brachythecium rutabulum*

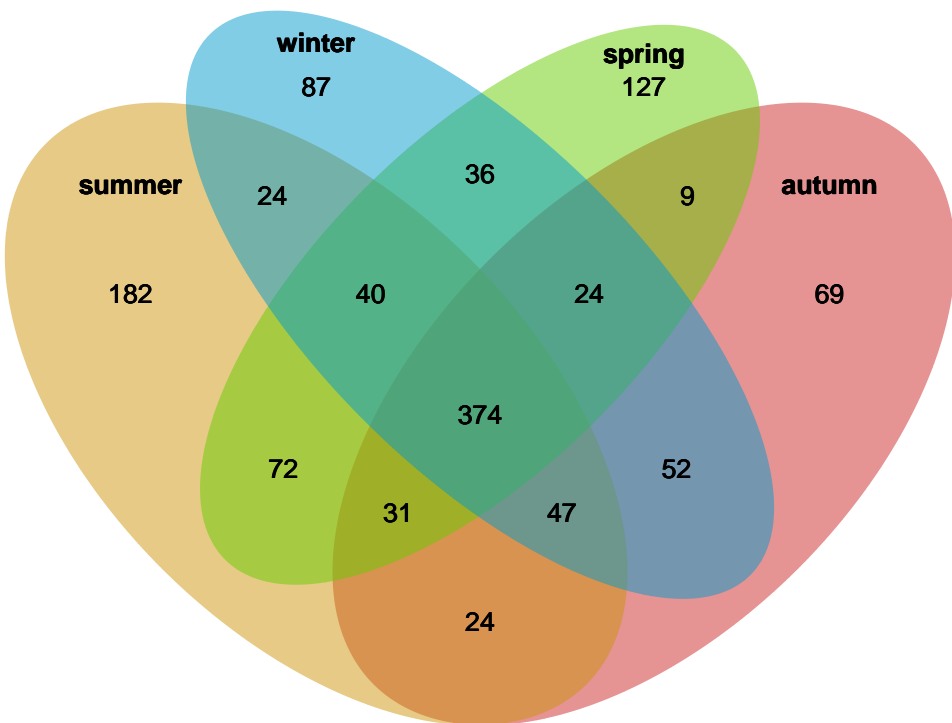

Supplement: Supplementary file 5 [file ECE3-8-9105-s005.pdf]

(e)

*Calliergonella cuspidata*

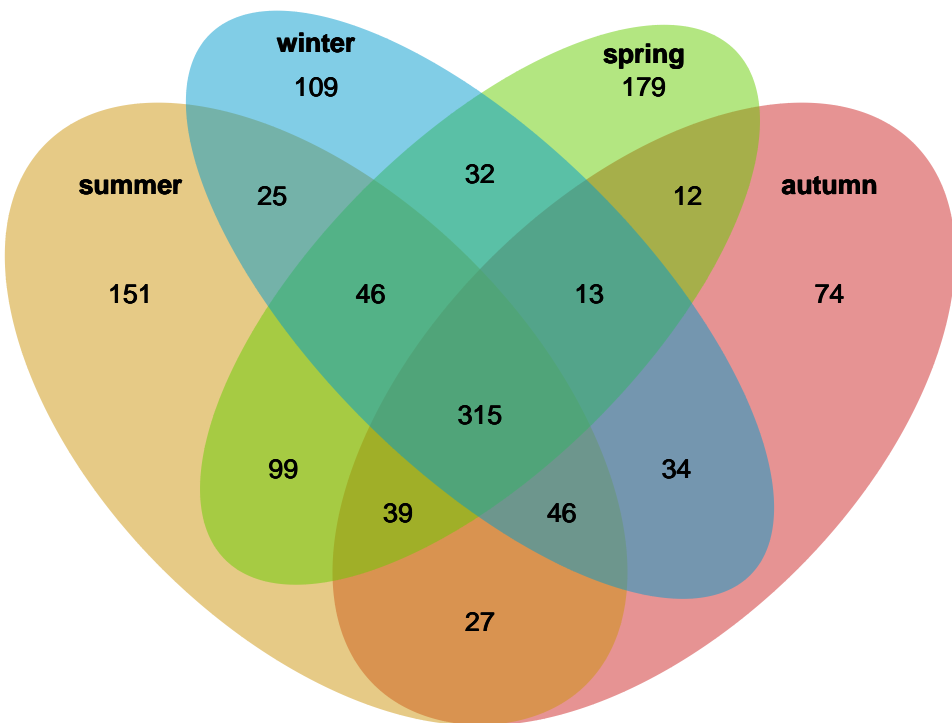

Supplement: Supplementary file 6 [file ECE3-8-9105-s006.pdf]

(f)

*Hypnum cupressiforme*

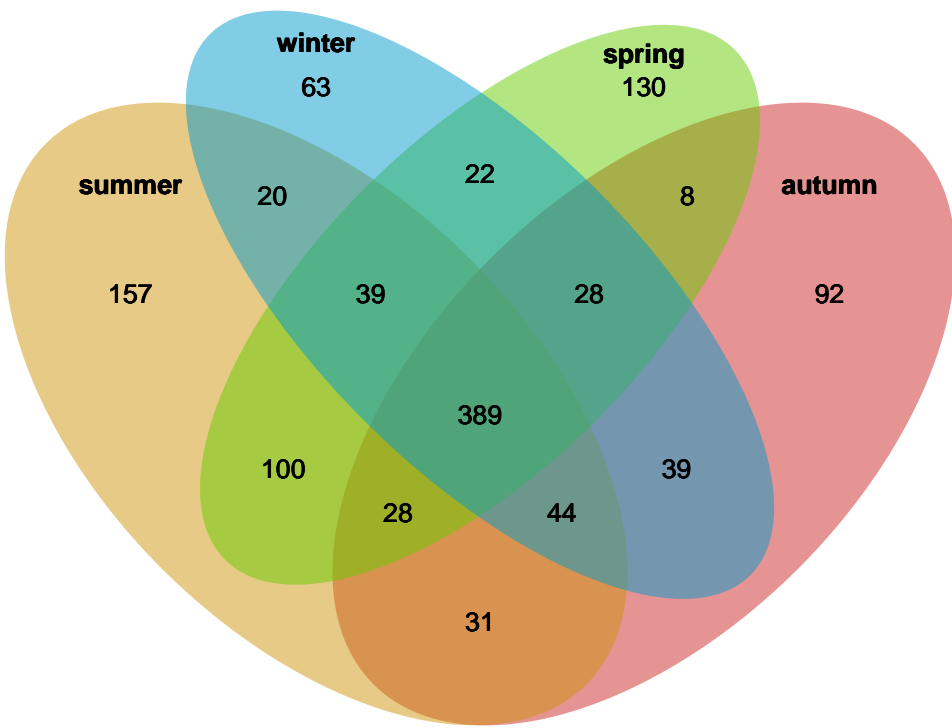

Supplement: Supplementary file 7 [file ECE3-8-9105-s007.pdf]

**(g)** *Rhytidiadelphus squarrosus*

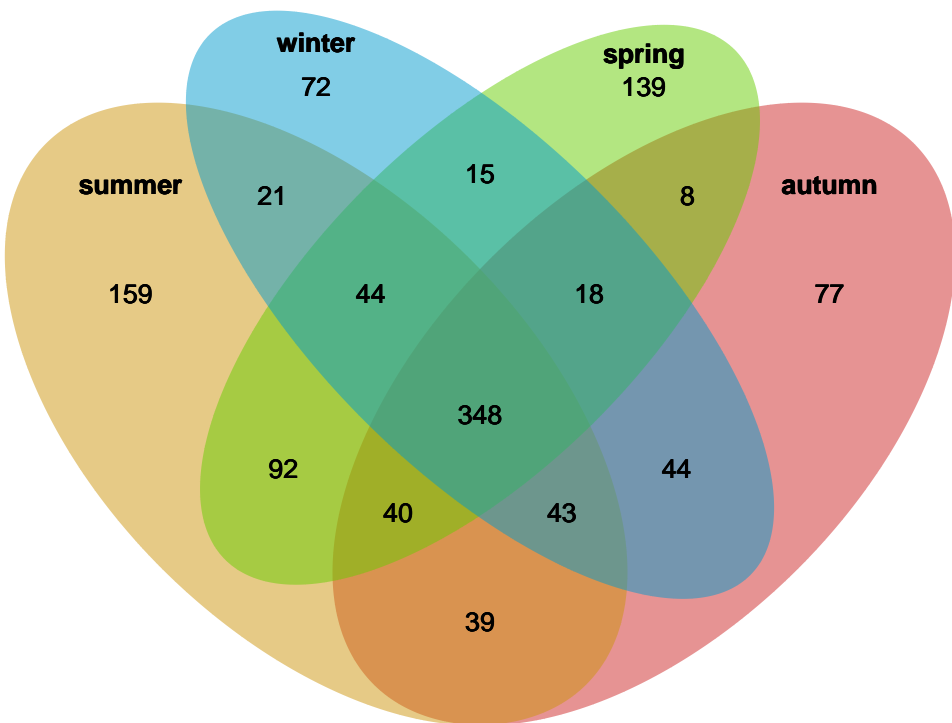

Supplement: Supplementary file 8 [file ECE3-8-9105-s008.pdf]

(h) *Fissidens taxifolius*

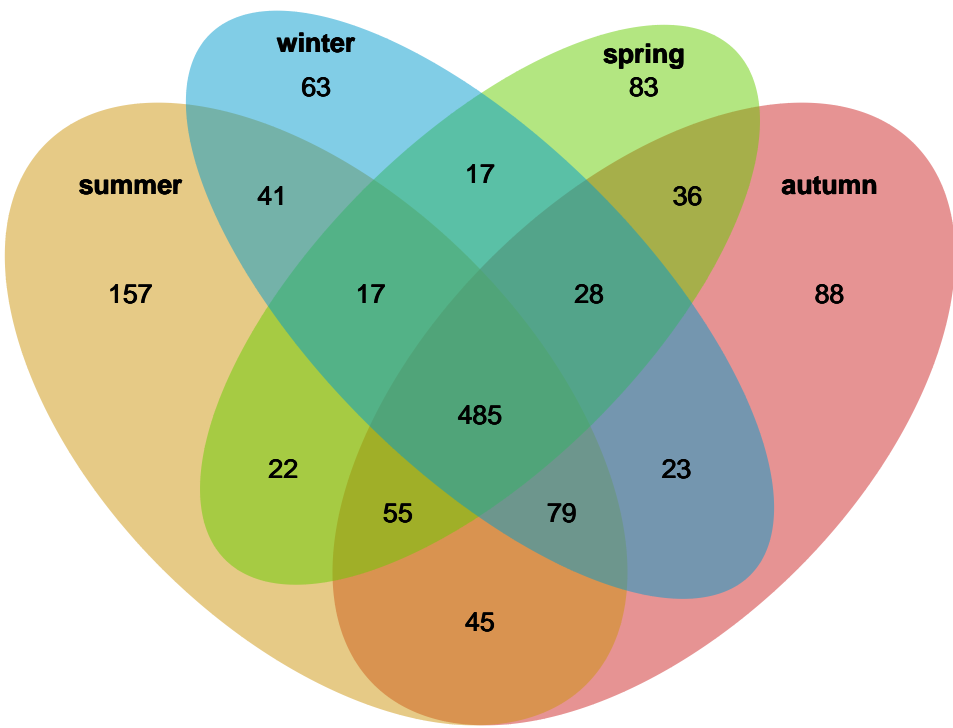

Supplement: Supplementary file 9 [file ECE3-8-9105-s009.pdf]

(i)

*Grimmia pulvinata*

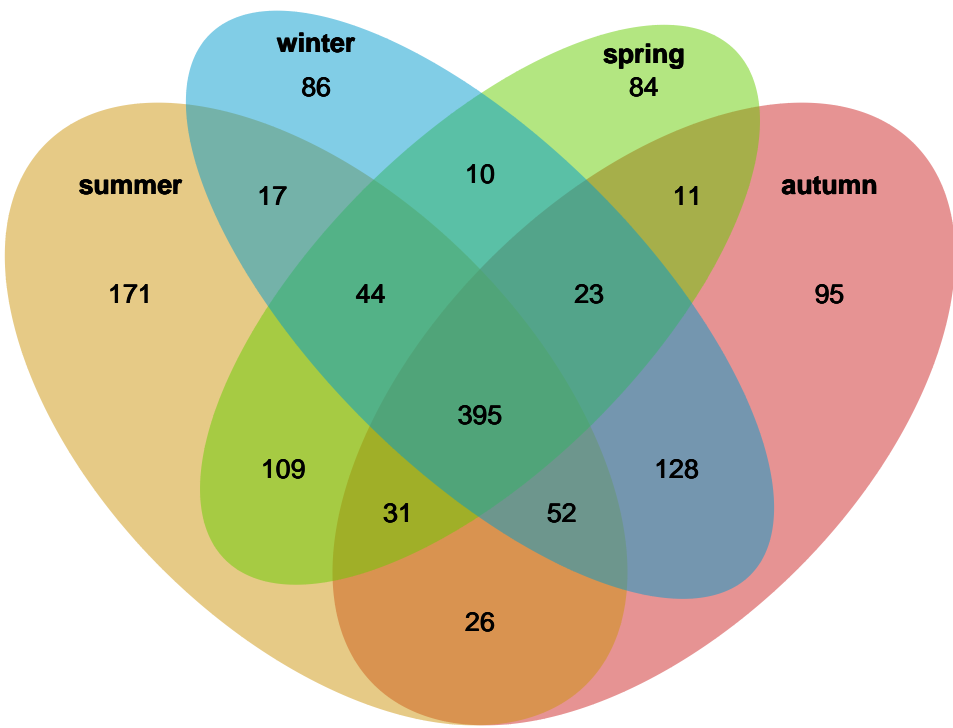

Supplement: Supplementary file 10 [file ECE3-8-9105-s010.pdf]

(j)

*Plagiomnium undulatum*

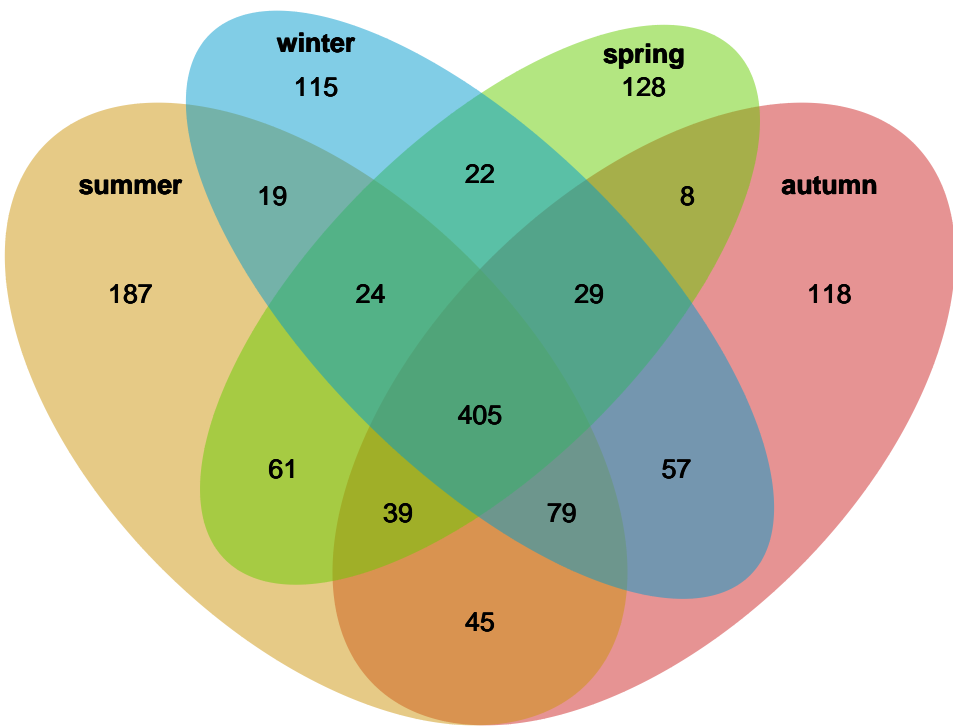

Supplement: Supplementary file 11 [file ECE3-8-9105-s011.pdf]

**(k)** *Polytrichum strictum*

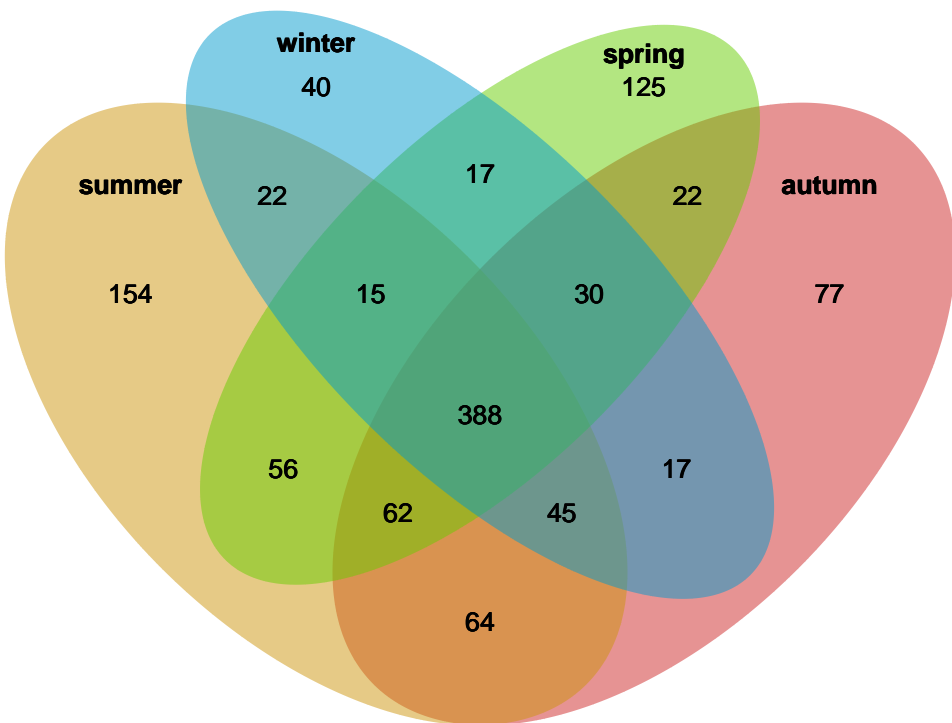

Supplement: Supplementary file 12 [file ECE3-8-9105-s012.pdf]

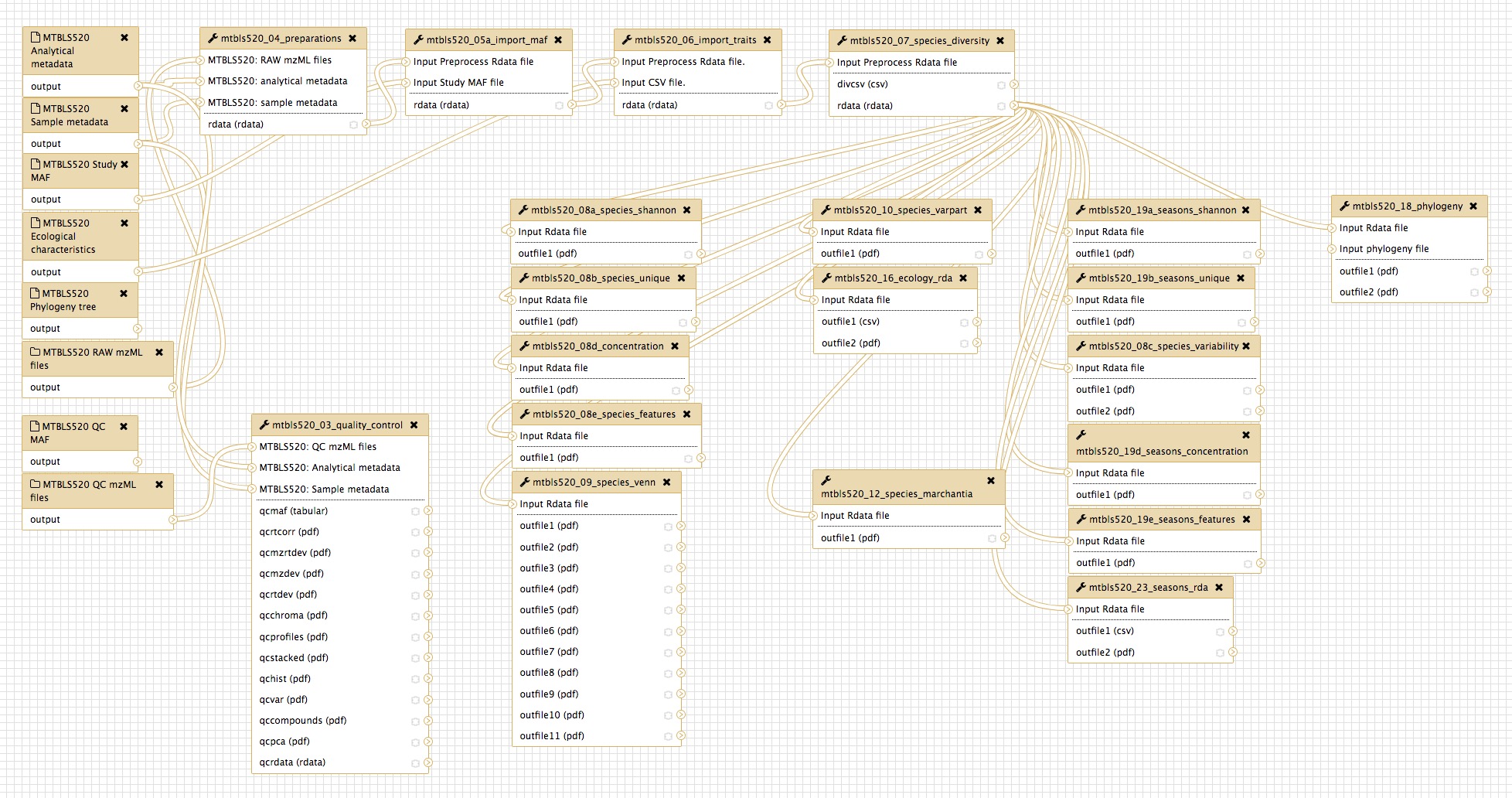

Supplement: Supplementary file 13 [file ECE3-8-9105-s013.jpg]

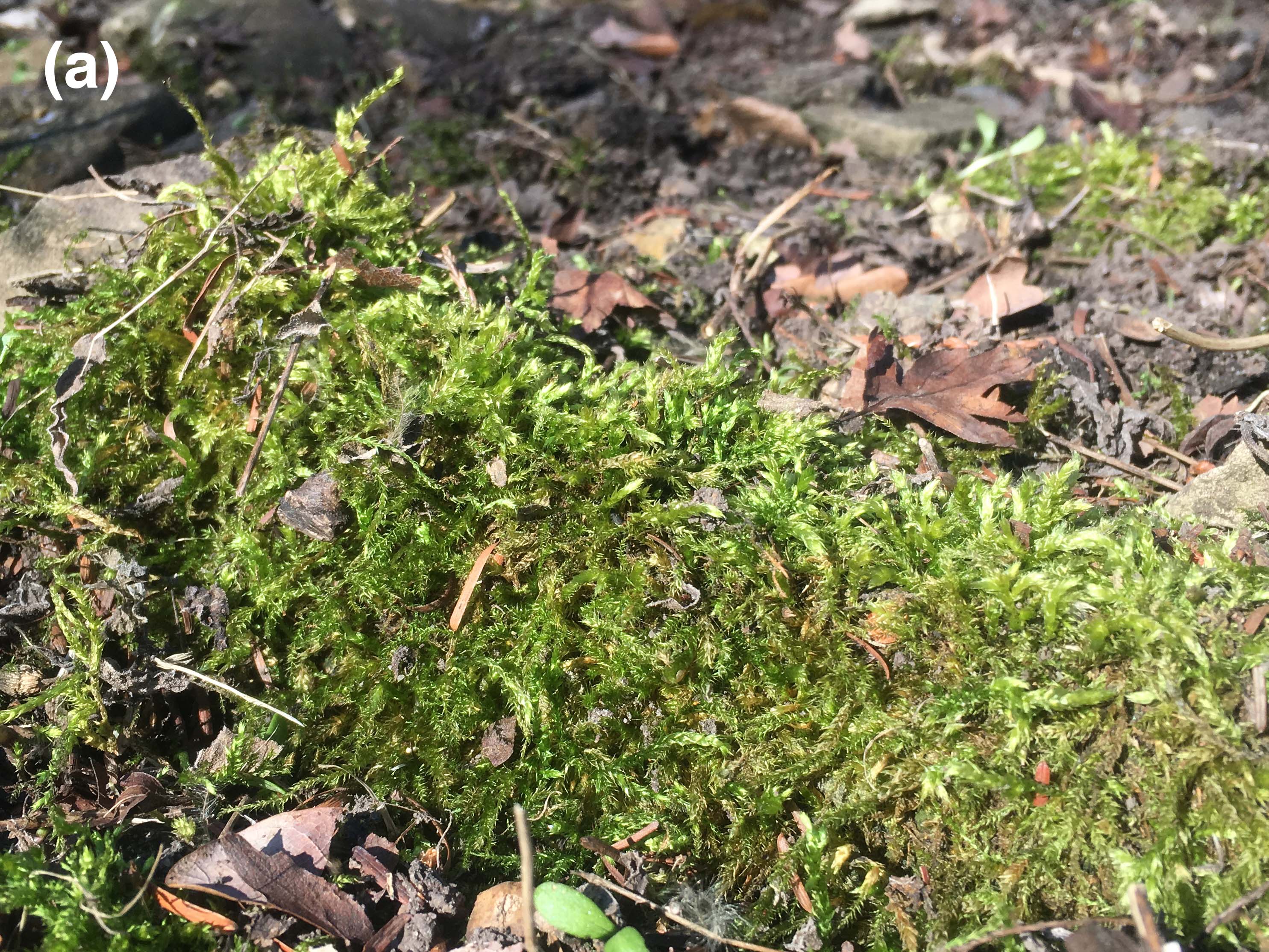

Supplement: Supplementary file 14 [file ECE3-8-9105-s014.jpg]

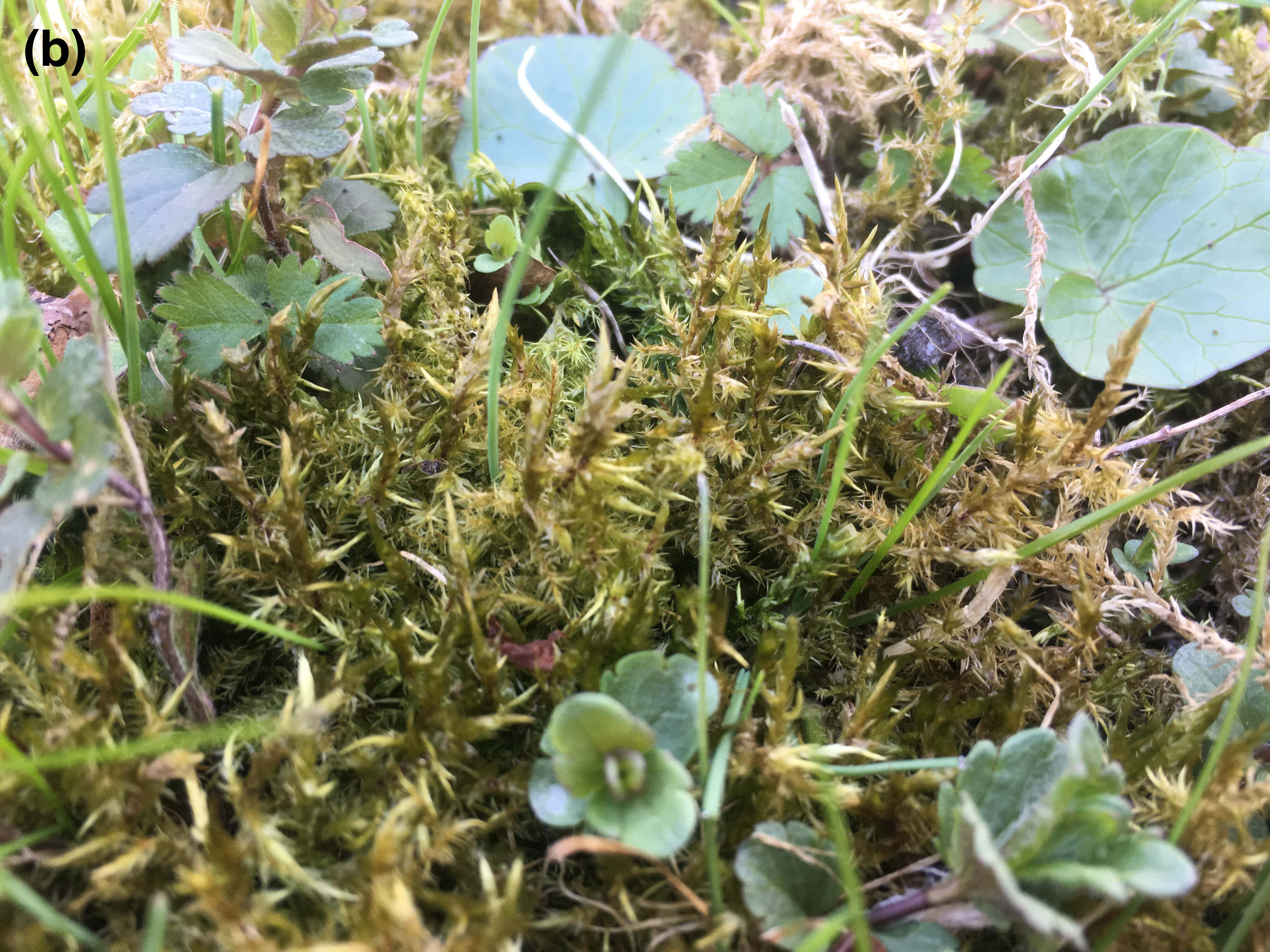

Supplement: Supplementary file 15 [file ECE3-8-9105-s015.jpg]

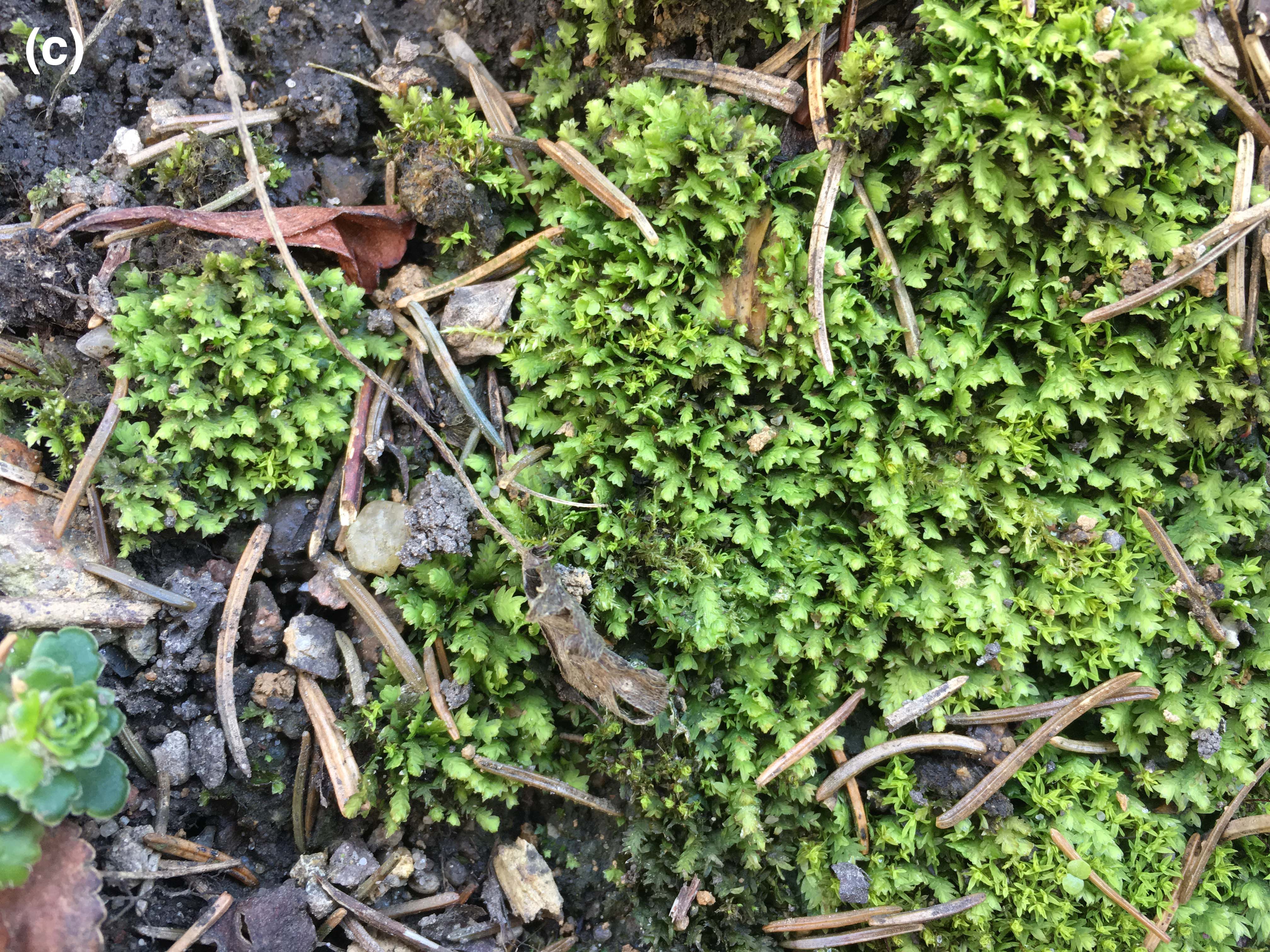

Supplement: Supplementary file 16 [file ECE3-8-9105-s016.jpg]

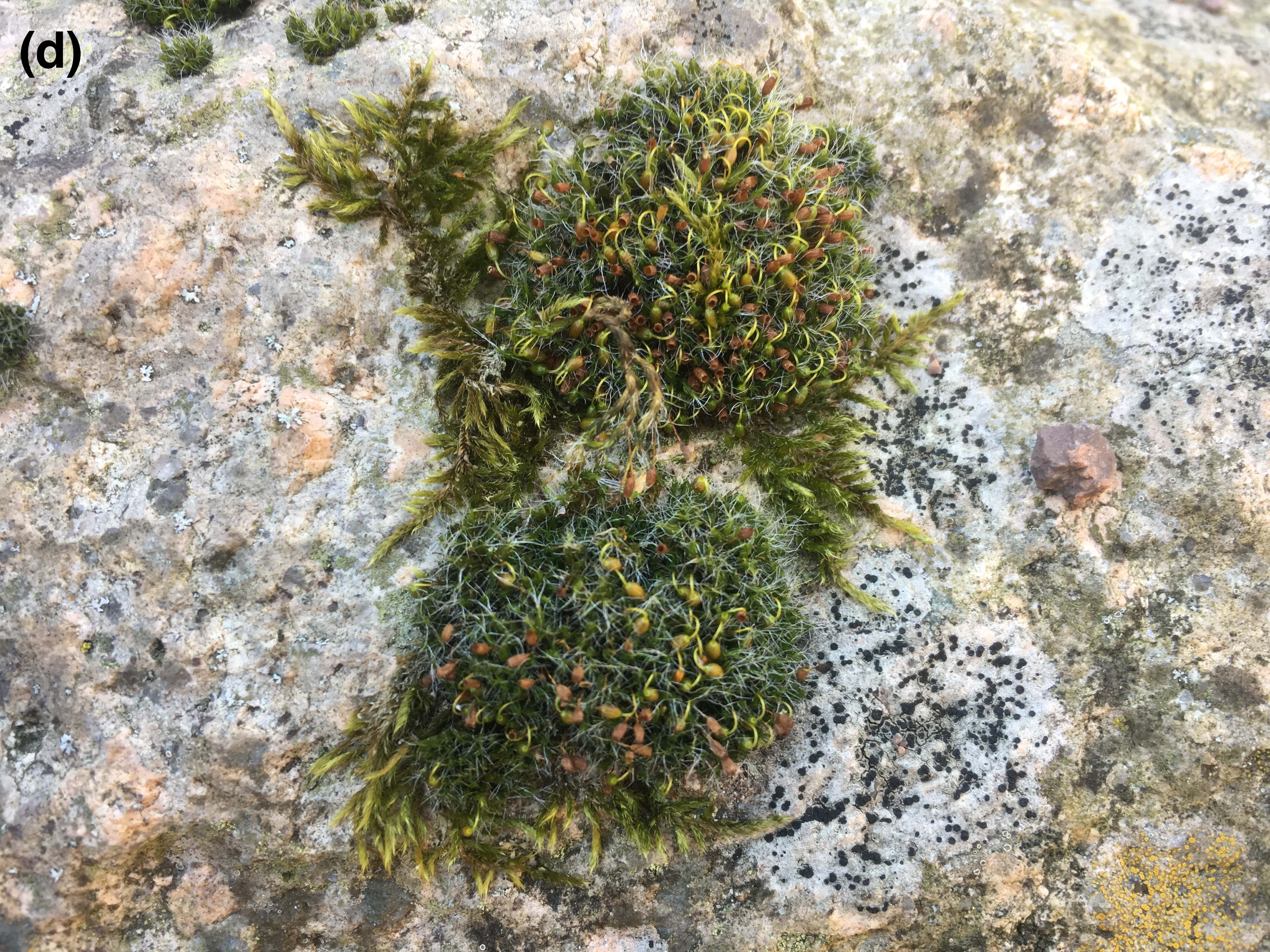

Supplement: Supplementary file 17 [file ECE3-8-9105-s017.jpg]

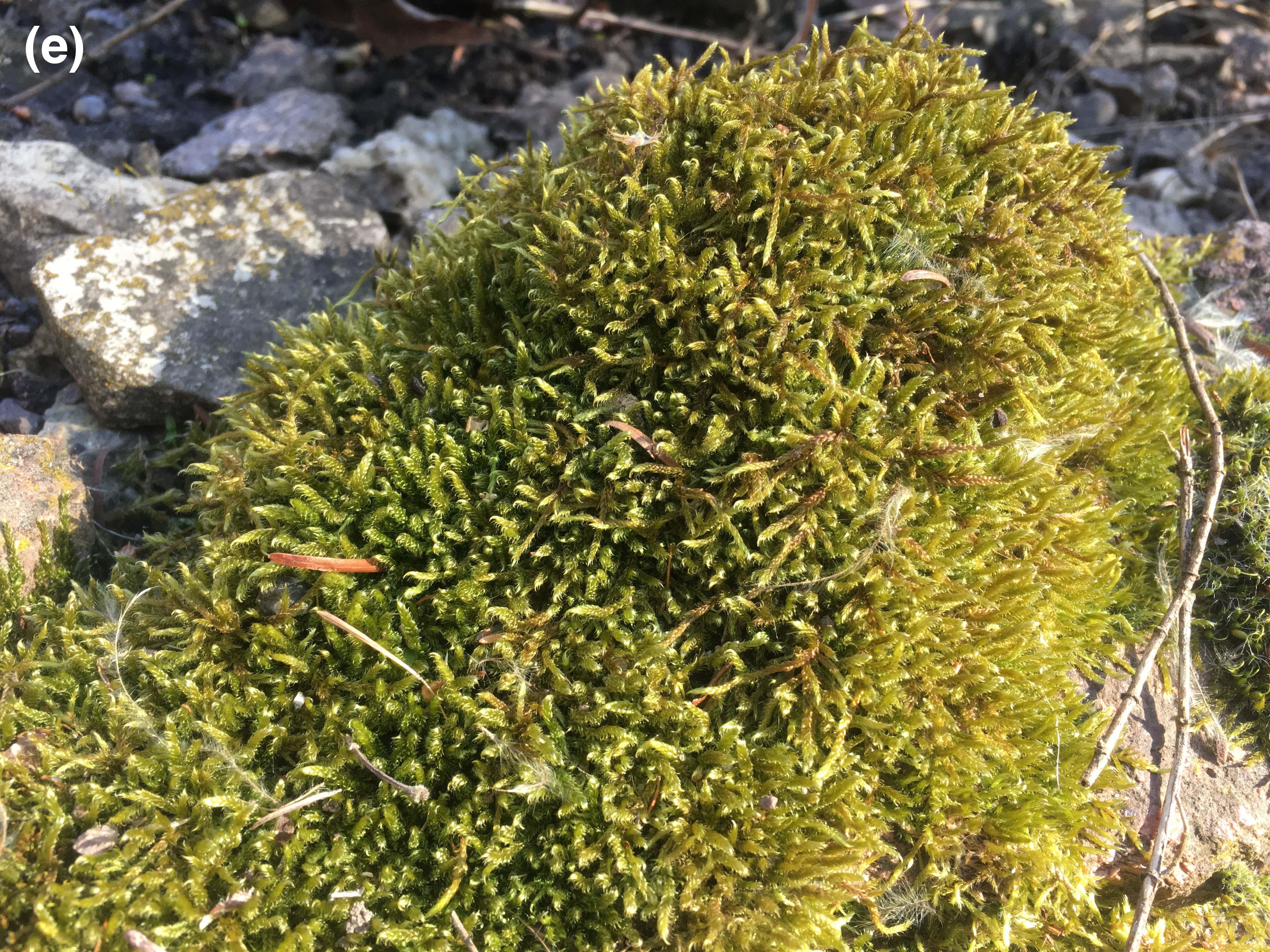

Supplement: Supplementary file 18 [file ECE3-8-9105-s018.jpg]

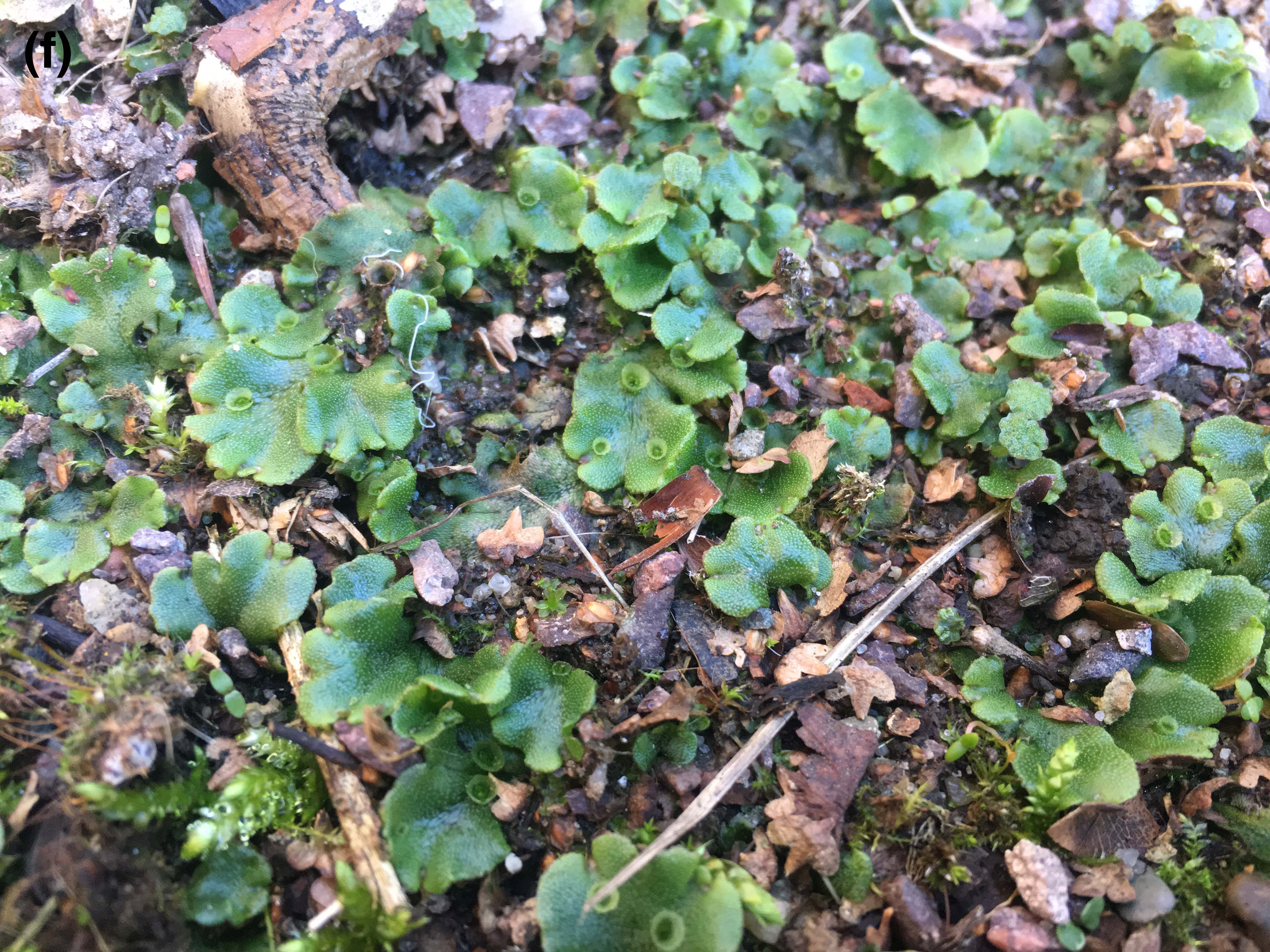

Supplement: Supplementary file 19 [file ECE3-8-9105-s019.jpg]

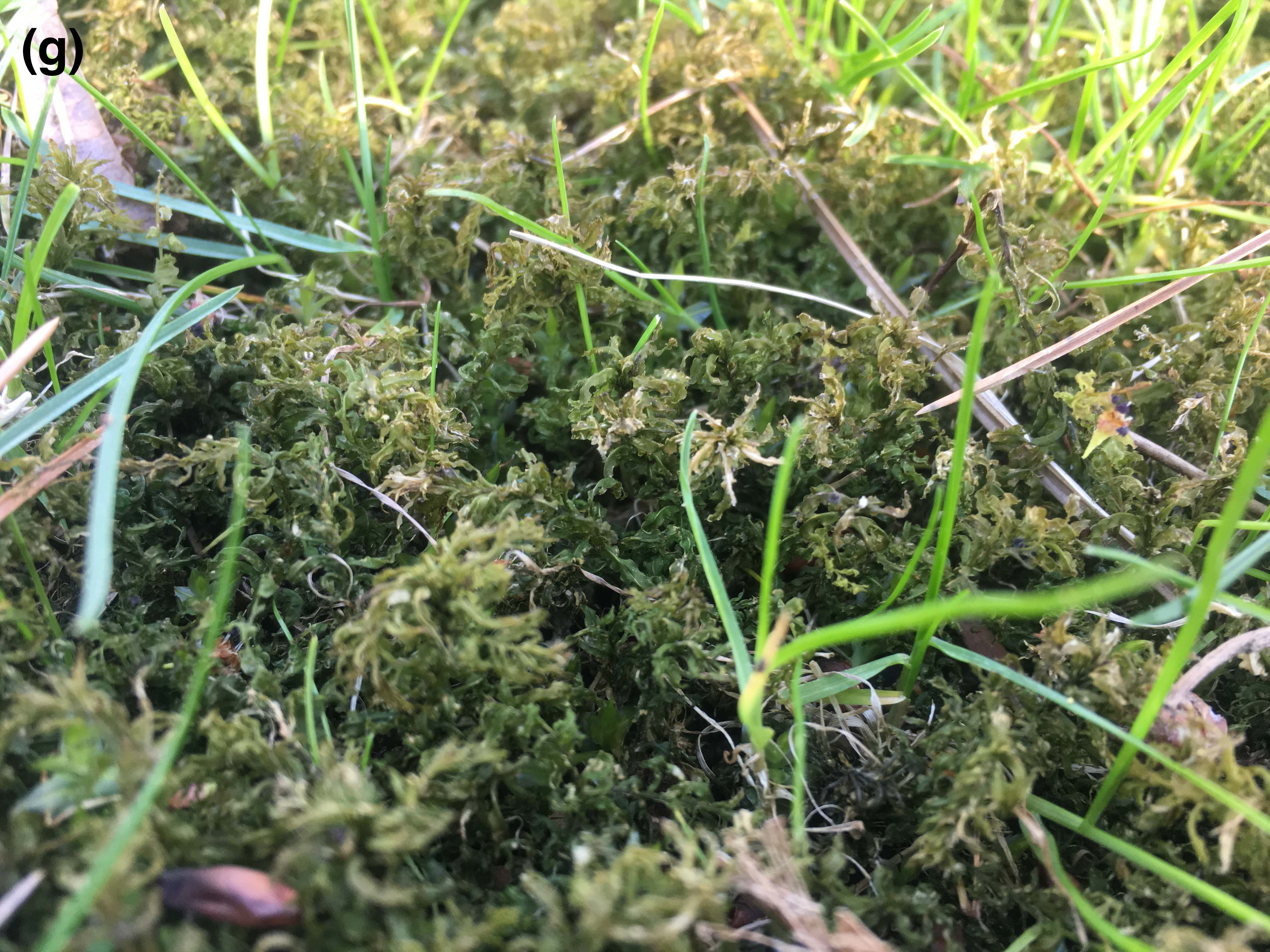

Supplement: Supplementary file 20 [file ECE3-8-9105-s020.jpg]

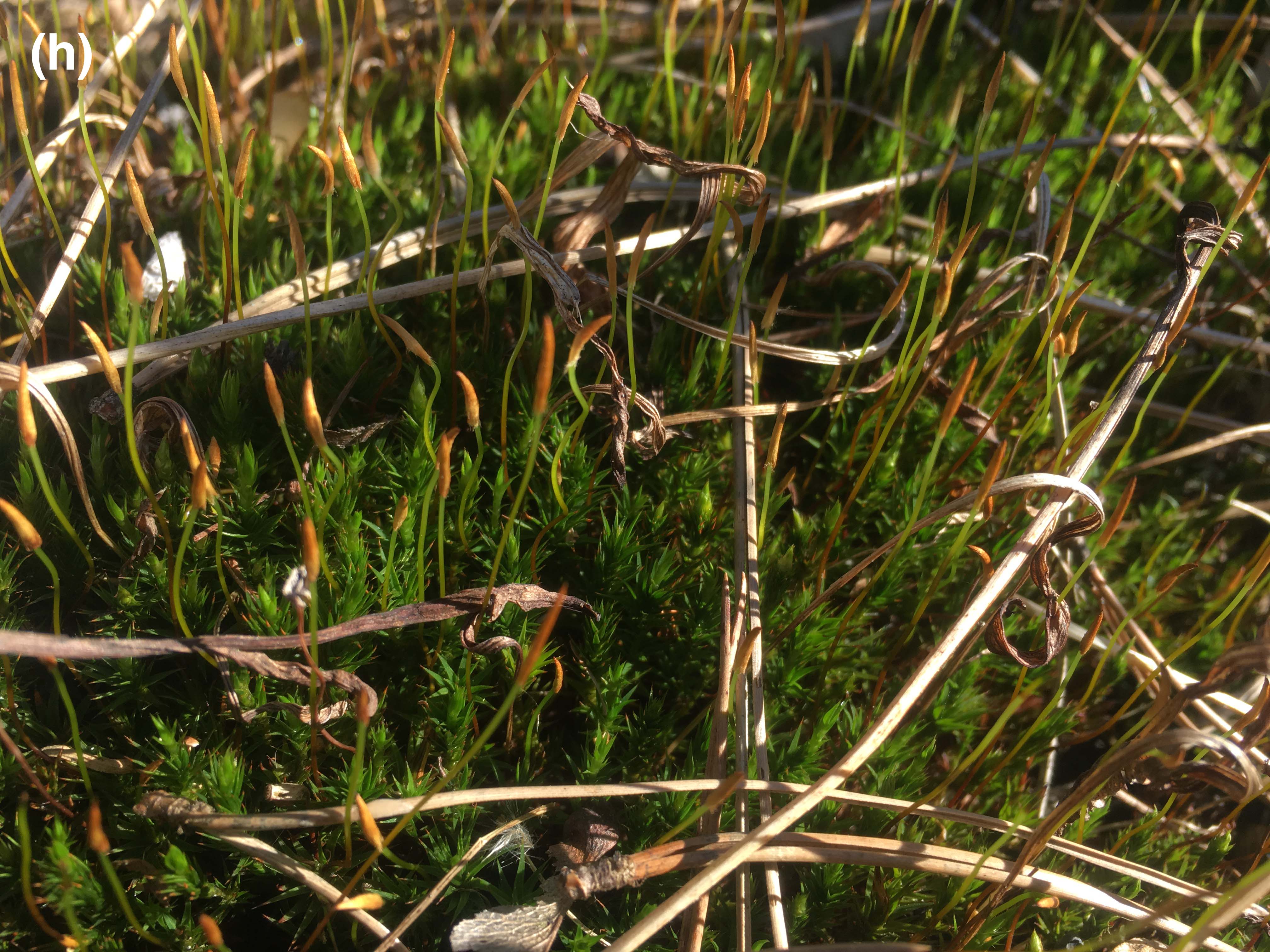

Supplement: Supplementary file 21 [file ECE3-8-9105-s021.jpg]

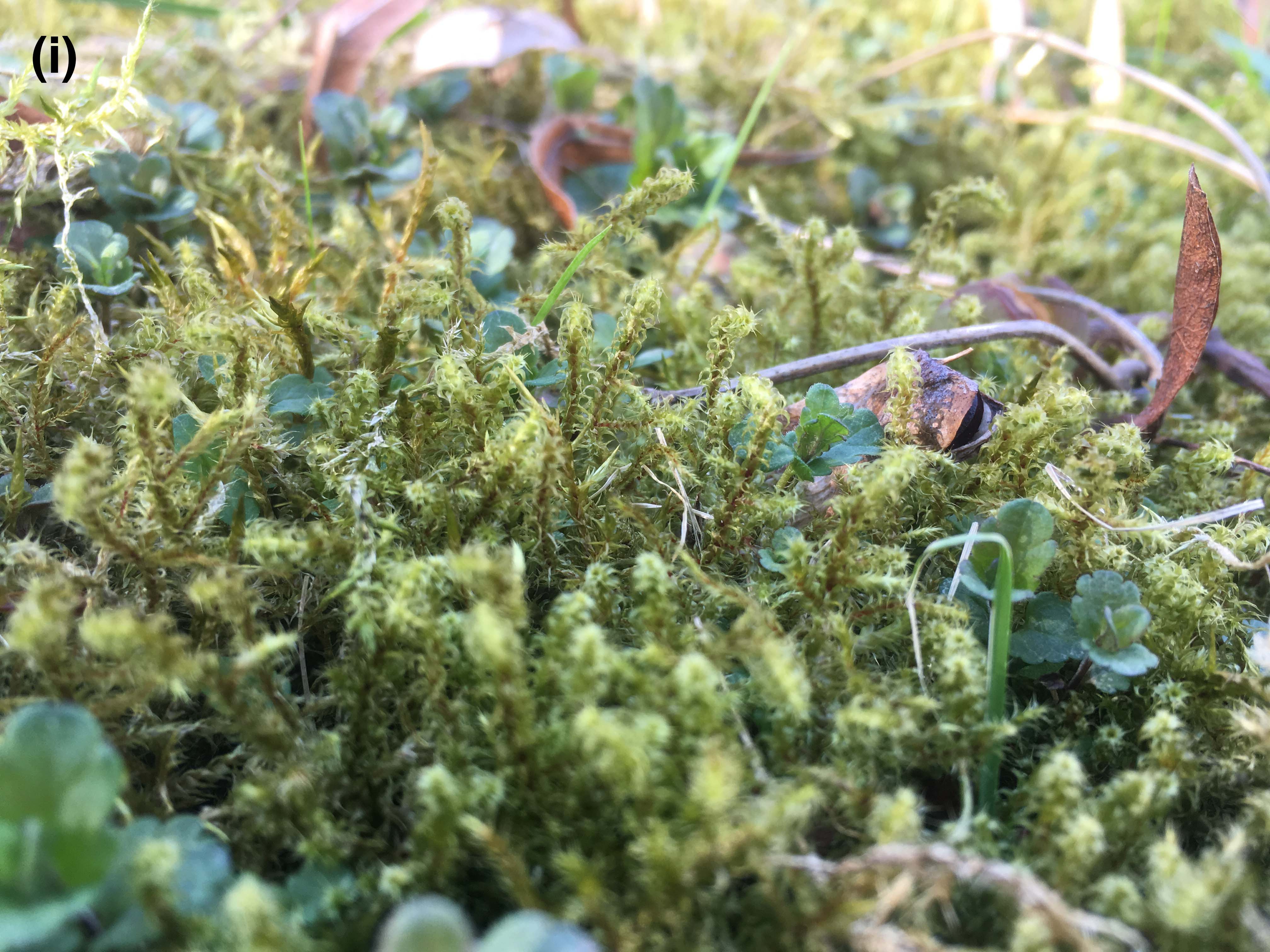

Supplement: Supplementary file 22 [file ECE3-8-9105-s022.jpg]
